# Heterogeneous selection dominated the temporal variation of the planktonic prokaryotic community during different seasons in the coastal waters of Bohai Bay

**DOI:** 10.1038/s41598-022-24892-1

**Published:** 2022-11-28

**Authors:** Wei Zhao, Liuyang Shi, Xingbiao Wang, Jingjing Wang, Song Xu, Lei Ma, Wanyu Zhu, Xiaoxia Zhang, Yifan Han, Zhiyong Huang

**Affiliations:** 1 Tianjin Key Laboratory of Industrial Biological Systems and Process Engineering, Tianjin Institute of Industrial Biotechnology, Chinese Academy of Sciences, Tianjin, China; 2 National Technology Innovation Center of Synthetic Biology, Tianjin, China

**Keywords:** Ecology, Microbiology, Environmental sciences, Ocean sciences

## Abstract

To explore temporal and spatial effects on the planktonic prokaryotic community composition (PCC) in the coastal region of the Bohai Sea, surface water samples were collected from 12  to  28 regularly distributed sites in Bohai Bay across 3 months from different seasons to characterize the PCC using high-throughput sequencing of the 16S rRNA V4 region. Prokaryotic α- and *β*-diversity showed significant temporal variation during the three sampling months. VPA analysis based on both weighted and unweighted UniFrac distances exhibited a shift of environmental and spatial effects on PCC variation with temporal variation. Quantification analysis of assembly processes on community turn over showed that “heterogeneous selection” dominated for PCC temporal variation, with basic abiotic parameters such as temperature, pH, ammonia nitrogen as the driving factors. Analysis of seasonal features showed that seasonal specific OTUs (ssOTUs) exhibited different seasonal attributions under the same phylum; meanwhile, the ssOTUs showed significant correlations with the driving environmental factors, which suggested that finer-level analysis was needed to more strictly reflect the temporal variation. Moreover, predicted nitrogen and sulfur metabolism were significantly shifted during the temporal variation. Our results clearly showed that seasonally varied environmental factors drive the “heterogeneous selection” process for PCC assembly in seawaters of Bohai Bay during different sampling seasons.

## Introduction

With the development and utilization of diverse molecular ecology techniques based on prokaryotic 16S rRNA gene sequences, it has become clear that marine microbial communities undergo temporal and spatial variations similar to most planktonic assemblages^1^. The taxonomic composition of microbial assemblages and their temporal and spatial dynamics in the oceans are likely to be of major importance in determining the role of microorganisms in marine biogeochemistry^2^. The biogeographic or temporally ‘predictable’ patterns in the marine environment have been studied in coastal regions around the world^3–9^. Seasonal patterns have been observed in the San Pedro Channel, Bermuda Atlantic Time Series station, Western English Channel time series station and Hawaii Ocean time series station^6^. These long time series studies mostly concentrated on a certain location, while the spatial distribution of the microbial community was often affected by distances from coasts and terrestrial inputs^7,10^. When considering both the spatial and temporal effects on the community variation, different results were observed at different sites. In the northern Adriatic Sea, high variability characterizes the spatial structure of bacterial assemblages, while scarce seasonality was found at all nine studied stations, and the assemblages were generally strongly affected by river inputs, especially in spring, when freshwater loads were higher^1^. Similarly, in coastal waters of the South China Sea, five regularly distributed sites in three seasons showed that the community diversity was better explained by spatial than by temporal patterns^8^. However, studies in Ofunato Bay (Pacific Ocean area of Japan)^11^ and Gosung Bay (South Sea, Korea)^9^ both showed that seasonal changes in environmental variables contribute to the dynamic structure of the bacterial community. It seems that the spatial and temporal distribution of marine microbial communities varies in different hydrological environments. Therefore, it is a challenge to draw a conclusion on how spatial and temporal factors determine the structuring of the microbial community in unexplored areas. Indeed, temporal factors are often considered to infer the environmental factors that change with time, such as temperature, DO, salinity and inorganic/organic resources, which inevitably change with the seasons. Therefore, rather than to say study the seasonal and spatial effect on microbial communities, it is better to say that it was to study the effects of environmental factors and spatial factors on microbial community over temporal variation.

When attempting to identify the key factors determining community variation, we need to confirm whether community variation was mainly influenced by deterministic or stochastic factors. Deterministic and stochastic processes influence the assembly of species into communities^12,13^. Deterministic processes are associated with ecological selection, in which abiotic and biotic factors determine the presence/absence and relative abundance of different species^14^. Stochastic processes include probabilistic dispersal and random changes in the relative abundance of different species (ecological drift) that are not caused by differences in environmentally determined fitness^15^. Deterministic and stochastic processes have been widely discussed in relation to various ecological processes, but little is known about their roles in the assembly of prokaryotic communities during natural temporal variation.

In addition, several studies have focused on the relationship between microbial community composition and ecosystem functioning^1,16,17^. Microbial communities are composed of functional groups (C and N fixers, ammonia oxidizers, sulfur reducers, etc.), so that the presence or absence of given organisms affects the biogeochemical activities occurring in a given environment, mainly by impacting elemental cycling rates^1^. Enrichment of sulfate-reducing bacteria was observed, and genes relevant for dissimilatory sulfate reduction were predicted in the coastal areas of the East China Sea^16^. The dynamics of nitrogen metabolism during the bloom process were also predicted, with different bacterial families contributing to the abundance of genes related to nitrogen metabolism^17^. However, the abundance of functional genes during different seasons in the coastal environment has rarely been researched, even though it may provide important information for understanding biogeochemical cycling. Moreover, changes in metabolic potential during different seasons also need to be taken into consideration.

This study was focused on Bohai Bay, China, which is located in the western region of the Bohai Sea in northern China. Based on the important geographical position and economic status of Bohai Bay, it has gradually become one of the intensive marine areas for marine scientific research. The spatial distribution of the bacterioplankton community in this area has been studied^18^, and it showed distinct spatial variation in the local environmental factors and dominant OTUs. Here, we selected 12~28 sites located at different geographic locations with different hydrological environments, among which several sites were located near the mouths of rivers, several sites were close to the tourist attractions, and several sites were at different distances from the shore. The main questions addressed by this study were as follows: (1) Do planktonic prokaryotic assemblages and functional potential show temporal patterns across different geographic locations? (2) How do environmental or spatial factors affect the PCC variation? 3) What processes drive the temporal or spatial pattern of the PCCs in the surface water of Bohai Bay?

## Results

### Variation in environmental parameters across space and time in Bohai Bay

**Figure 1:**
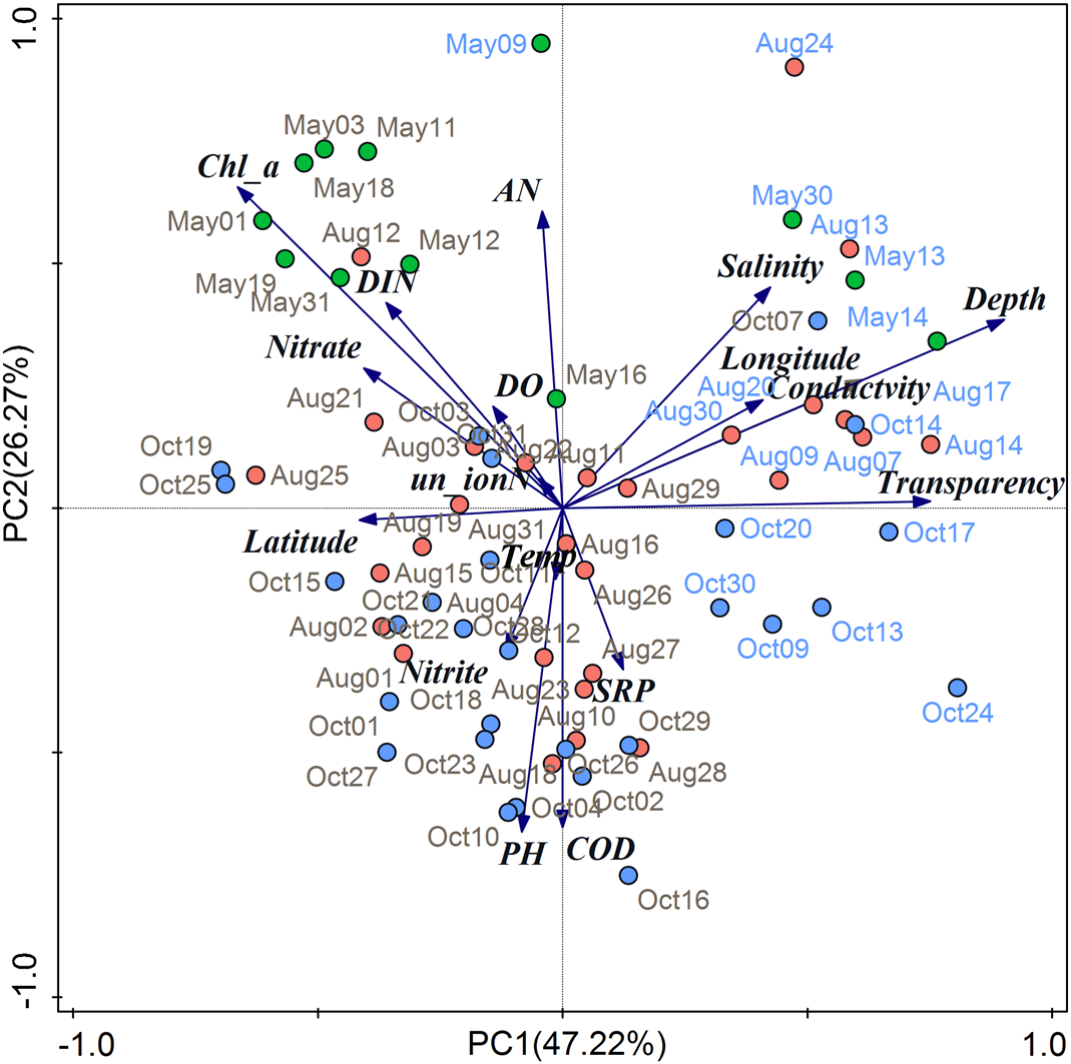
Biplot of the principal component analysis (PCA) for environmental parameters in the seawater samples of the Bohai Bay coastal area across different seasons and sites. The two principal components (PC1 and PC2) explained 73.49% of the total variation in the environmental data and showed clear partitioning of offshore samples (in blue font) from other nearshore samples along PC1 and partitioning of May samples from August and October along PC2. The variables transparency and latitude were strongly correlated with PC1, and the variables ammonia nitrogen (AN), COD, pH, soluble reactive phosphate (SRP), and nitrite were strongly correlated with PC2. Chlorophyll a (*Chl*_a), dissolved inorganic nitrogen (DIN), nitrate and DO were mainly positively correlated with samples from May, while salinity, longitude, depth and conductivity were mainly positively correlated with offshore samples. Blue arrows represent environmental parameters, and circles in color represent sampling points.

The environmental parameters of samples collected near the Tianjin coastal area from different stations and seasons exhibited high temporal and spatial heterogeneity. The seawater temperature was 28.09 ± 0.53 °C in Aug, 17.48 ± 2.36 °C in May, and 19.55 ± 1.26 °C in Oct (Table 1). The seasonal variation in seawater temperature corresponded to the meteorological characteristics in Bohai Bay, with warm seawater in summer and relatively cool seawater in spring. The salinity was 29.69 ± 2.71‰ in Aug, 33.19 ± 0.33‰ in May, and 30.15 ± 1.63‰ in Oct. Seasonal variations in salinity may be mainly related to freshwater loading. According to the precipitation observed data of Bohai Bay in previous years, the rainfall amount and days in summer are the most^19^, which may lead to the increase in runoff and the relatively low salinity in summer. Chlorophyll a (Chl a) was highest in May, with lower levels in Aug and Oct. The dissolved inorganic nitrogen (DIN) was significantly higher in May and Aug than in Oct. The higher level of DIN in May and Aug may be related to terrestrial input and supply for the demand of phytoplankton growth. In October, the temperature and DIN content were both not suitable for phytoplankton growth, and *Chl*_a showed the lowest value. Spatially, the DIN distribution across the three seasons was rather similar, with high values observed in nearshore waters and low values in offshore waters (Dataset S1 & Fig. S1), which suggested that terrestrial input was an important source of DIN. The pH, soluble reactive phosphate (SRP) and chemical oxygen demand (COD) showed relatively higher values in October than in August and May, which may be caused by the dead phytoplankton release and terrestrial loadings through coasts and rivers. The dissolved oxygen (DO), conductivity and depth did not show significant variation among sampling times (Table 1), while the conductivity and depth had relatively higher values at offshore stations (Dataset S1) since the more remote the sampling water was, the greater the depth was in Bohai Bay and the closer it was to the open sea with higher salinity and conductivity. The ordination plot showed distinct partitioning of samples from nearshore and offshore sites along principal component axis 1 (PC1) (Fig. 1). The ordination plot could explain 73.49% of the total variation in the geo-physical–chemical parameters and revealed a linear positive correlation between different parameters (Fig. 1). AN, DIN, nitrate and *Chl*_a were most crucial in the partitioning of samples from May and the other 2 months; salinity, longitude, depth and conductivity were crucial for the partitioning of samples from offshore and nearshore stations; pH, COD, SRP, nitrite and temperature were crucial for the partitioning of samples from nearshore stations in August and October and samples from offshore stations. Overall, the principal component analysis (PCA) plot clearly showed both the temporal and spatial variation of the measured environmental parameters, indicating that complex biogeochemical processes and hydrodynamic conditions lead to the variation among sites and seasons.

**Table 1:** The independent-samples *t* test of environmental variables and α-diversity among different months.

	May	Aug	Oct
Temp/°C	17.48 ± 2.36^c^	28.09 ± 0.53^a^	19.55 ± 1.26^b^
Salinity/‰	33.19 ± 0.33^a^	29.69 ± 2.71^b^	30.15 ± 1.63^b^
Conductivity/ms/cm	47.04 ± 0.92^a^	45.92 ± 2.44^a^	46.49 ± 1.13^a^
DO/mg/L	8.21 ± 0.51^a^	7.58 ± 1.51^a^	7.93 ± 0.39^a^
PH	8.04 ± 0.07^b^	8.39 ± 0.09^a^	8.38 ± 0.07^a^
SRP/mg/L	0.003 ± 0.002^c^	0.01 ± 0.004^b^	0.02 ± 0.01^a^
COD/mg/L	0.3 ± 0.14^c^	1.23 ± 0.82^b^	2.45 ± 0.49^a^
Nitrite/mg/L	0.02 ± 0.01^b^	0.05 ± 0.04^a^	0.04 ± 0.02^a^
Nitrate/mg/L	0.24 ± 0.08^a^	0.21 ± 0.09^a^	0.16 ± 0.07^b^
AN/mg/L	0.13 ± 0.02^a^	0.1 ± 0.02^b^	0.02 ± 0.02^c^
DIN/mg/L	0.39 ± 0.08^a^	0.36 ± 0.1^a^	0.23 ± 0.07^b^
un_ionN/mg/L	0.004 ± 0.001^b^	0.01 ± 0.003^a^	0.001 ± 0.001^c^
Depth/m	8.31 ± 6.25^a^	7.71 ± 6.01^a^	6.98 ± 5.39^a^
Trans/cm	0.79 ± 0.5^b^	1.4 ± 0.65^a^	1.2 ± 0.58^a^
*Chl*_a/μg/L	4.84 ± 2.76^a^	2.37 ± 1.77^b^	2.24 ± 1.74^b^
Faith_pd	58.28 ± 12.75^b^	67.99 ± 16.77^a^	44.36 ± 3.68^c^
OTU_richness	863.75 ± 211.61^b^	985.37 ± 256.73^a^	675.01 ± 58.14^c^
Shannon	7.37 ± 0.44^c^	7.91 ± 0.69^a^	7.67 ± 0.28^b^
Eveness	0.76 ± 0.03^c^	0.8 ± 0.04^b^	0.82 ± 0.02^a^

Data in the table are the Means ± SD; The different normal letters (abc) in the same row indicate significant difference among sampling times at 0.05 level (n > 3), if they contain the same letter it means the difference is not significant, if they contain different letter it means the difference was significant. Temp, water temperature; DO, dissolved oxygen; SRP, soluble reactive phosphates; COD, chemical oxygen demand; AN: ammonia nitrogen; DIN, dissolved inorganic nitrogen; Trans, transparency; un_ionN, nonionic ammonia; *Chl*_a, chlorophyll a.

### Prokaryotic α/*β*-diversity variation

**Figure 2:**
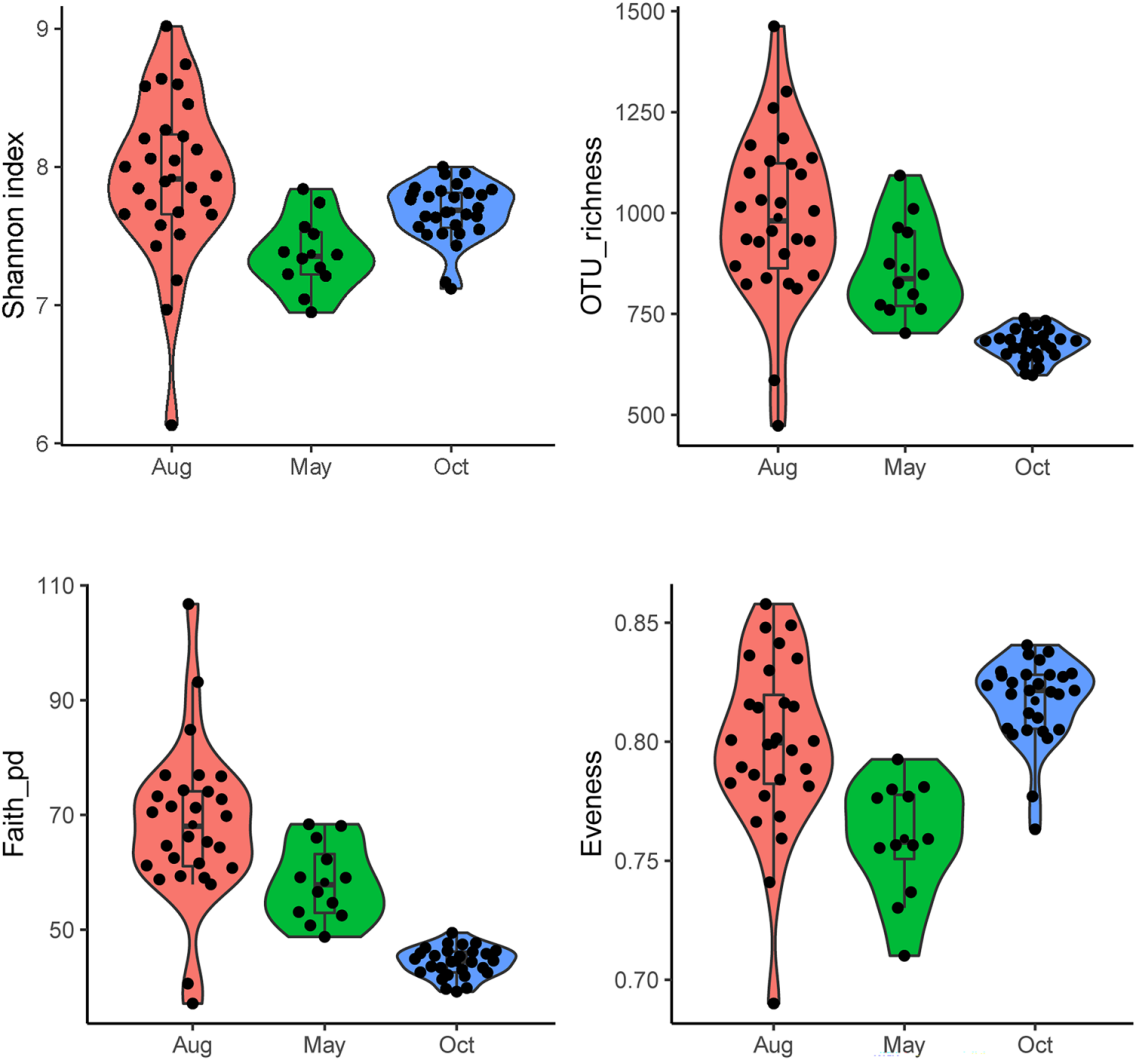
Alpha diversity of shannon, eveness, faith_pd (phylogenetic diversity) and OTU richness value of the prokaryotic community of all the samples from different stations at different sampling times.

**Figure 3:**
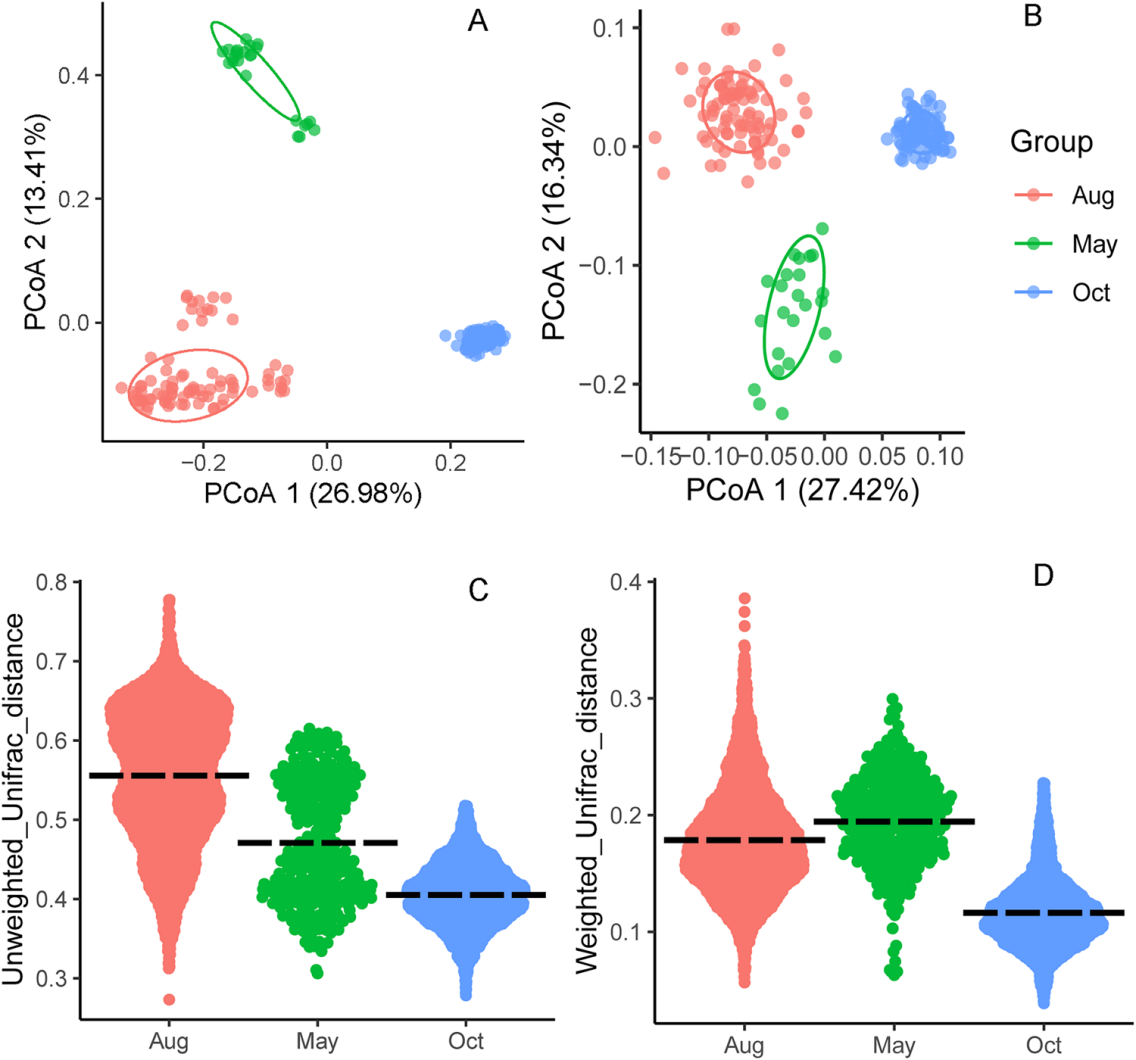
Principal coordinate analysis (PCoA) based on unweighted (**A**) and weighted (**B**) UniFrac distances for prokaryotic communities in the surface waters; box plots showing the unweighted (**C**) and weighted (**D**) UniFrac distances among each station at different sampling times.

Measures of *α-*diversity showed significant differences in shannon, evenness, faith_pd and OTU richness between samples from May/Aug and Oct (Fig. 2, Table 1). Principal coordinates analysis (PCoAs) based on weighted UniFrac (WUF) distance and unweighted UniFrac (UUF) distance showed that the PCC from different sampling months separated across the first and second principal coordinates (Fig. 3A-B). Both the analysis of similarity (ANOSIM) and permutational multivariate analysis of variance (PERMANOVA/ADONIS) results indicated that the prokaryotic communities varied significantly across different sampling months when using a WUF distance metric (ANOSIM, r = 0.709, *P* = 0.001; ADONIS, R^2^ = 40.0%, *P* = 0.001) and UUF distance metric (ANOSIM, r = 0.934, *P* = 0.001; ADONIS, R^2^ = 38.7%, *P* = 0.001). At the same time, the prokaryotic α– and *β*-diversity both showed high within-month variability in Aug (Figs. 2, 3C–D), which indicated that the community varied greatly among different sites in Aug.

### Correlation between prokaryotic α/*β*-diversity and physical, chemical and geographic factors

The *α-*diversity measurements exhibited significant positive correlations with temperature, pH, SRP, AN and un_ionN (Dataset S2). The correlation between *α-*diversity indexes and geo factors (longitude and latitude) was not strong or significant both in samples across the three sampling times or from each sampling time (Dataset S2).

The environmental variation significantly correlated with *β*-diversity among the three seasons (r_weighted = 0.4558, r_unweighted = 0.4631, *P* = 0.001, Table 2), with pH, AN, temperature, un_ionN, COD, nitrite, SRP, salinity, DO and DIN as the main individual determinants. However, it did not show significant correlations with *β*-diversity at any sampling time except in Oct (Table S1).

**Table 2:** Spearman’s rank correlation between environmental/spatial variability (Euclidean distance) and prokaryotic β-diversity (weighted/unweighted UniFrac distance) among all samples from different season.

Variables	Weighted					Unweighted				
	**r**	**p**	**r_**_**temporal**_	**p_**_**temporal**_	**N**	**r**	**p**	**r_**_**temporal**_	**p_**_**temporal**_	**N**
pH	**0.4392**	0.001	0.05697	0.144	68	**0.5104**	0.001	− 0.1392	1	68
AN	**0.4939**	0.001	**0.1403**	0.004	68	**0.5093**	0.001	**0.1922**	0.001	68
Env	**0.4558**	0.001	**0.1964**	0.002	68	**0.4631**	0.001	**0.2551**	0.001	68
Temp	**0.4819**	0.001	− 0.0596	0.854	68	**0.5491**	0.001	− 0.004416	0.554	68
un_ionN	**0.3849**	0.001	0.0199	0.403	68	**0.4657**	0.001	**0.2675**	0.001	68
COD	**0.3587**	0.001	**0.1043**	0.007	68	**0.3903**	0.001	**0.1848**	0.001	68
Nitrite	**0.2518**	0.001	0.155	0.098	68	**0.1887**	0.002	**0.1229**	0.01	68
SRP	**0.133**	0.009	− 0.05007	0.803	68	**0.1911**	0.002	**0.09605**	0.026	68
Salinity	**0.09235**	0.046	− 0.02944	0.653	68	**0.1855**	0.001	**0.0967**	0.02	68
DO	**0.1653**	0.014	0.06852	0.201	68	**0.2042**	0.001	**0.2661**	0.001	68
DIN	**0.2157**	0.002	**0.1239**	0.031	68	**0.173**	0.001	**0.09299**	0.035	68
Chl_α	0.09864	0.076	− 0.03354	0.688	68	**0.1867**	0.001	− 0.04271	0.839	68
Nitrate	0.1007	0.061	0.092	0.09	68	0.05499	0.114	0.01883	0.289	68
Depth	0.0925	0.071	0.1442	0.018	68	− 0.04877	0.823	0.002255	0.421	68
Trans	0.1017	0.019	**0.1477**	0.022	68	0.01094	0.35	− 0.02779	0.732	68
Geo	0.03149	0.293	0.03907	0.295	68	− 0.005656	0.491	0.03601	0.233	68
Longitude	0.02462	0.331	0.0302	0.327	68	− 0.02081	0.644	0.013	0.379	68
Latitude	− 0.0125	0.603	0.01818	0.326	68	0.0194	0.25	**0.08091**	0.014	68
Conductivity	− 0.00484	0.503	0.06671	0.176	68	− 0.00338	0.473	**0.1746**	0.002	68

r, correlation coefficients between environmental/spatial variability and prokaryotic community distance derived from Mantel testes with 999 permutations, r__temporal_, correlation coefficients between environmental/spatial variability and prokaryotic community distance controlled by temporal variation derived from partial Mantel testes with 999 permutations. Data in bold indicate significant correlations (*P* < 0.05). Abbreviations: Env, environmental distance (Euclidean distance); Geo, geographic distance. Refer to Table 1 for other abbreviations.

The geographic distance was not correlated with prokaryotic *β*-diversity (variation in community composition; r < 0.03, *P* > 0.05; Table 2) among the three sampling times. However, samples from Aug and Oct exhibited a significant correlation between *β*-diversity and geographic distance (Table S1).

### Factors driving the PCC variation

PERMANOVA using the UUF/WUF distance indicated that temperature variation explained the largest part of community variation among the investigated factors (34.90%/19.83%, *P* = 0.001, Dataset S3), with AN (31.84%/13.56%, *P* = 0.001) and salinity (12.91%/6.21%, *P* = 0.001) as the second and third most significant sources of variation.

The variance partitioning analysis (VPA) conducted on both UUF/WUF distances showed that almost 100% percent of the variation in PCC among all three sampling times was explained by the detected environmental factors. In May, no environmental or spatial factors could be selected as significantly explain the PCC variation; in Aug, the joint effects of environmental and spatial factors could explain 49.5% of the variation; in Oct, based on WUF distance, the spatial factors could purely explain 10.5%, environmental factors could purely explain 38.8%, their joint effects could explain 28.2%, and based on UUF distance, the joint effects of environmental factors and trend could explain 13.7% of the PCC variation. These results indicated dramatic shifts in the spatial or environmental factor effects on the PCC variation at different sampling times in Bohai Bay (Table 3).

**Table 3:** Variance partitioning analysis of prokaryotic community in Bohai Bay according to seawater environmental factors and geospatial factors. The spatial factors including linear trend and PCNM variables. Forward selection procedures were used to select the best subset of environmental, trend, and PCNM variables explaining community variation, respectively. The community variation was calculated on the weighted and unweighted UniFrac distance matrix, respectively. Monte Carlo permutation test was performed on each set without the effect of the other by permuting samples freely (999 permutations).

	Env	Trend	PCNM	Trend*Env	Env*PCNM	Trend*PCNM	Trend*Env*PCNM
**May**							
Weighted	❌	❌	❌	❌	❌	❌	❌
Unweighted	❌	❌	❌	❌	❌	❌	❌
**Aug**							
Weighted	0.114 (0.051)	0.019 (0.277)	0 (0.411)	0.082	0.067	0.078	0.095
Unweighted	0.082 (0.073)	0 (0.752)	0.053 (0.095)	0.032	0.189	0.112	0.162
**Oct**							
Weighted	0.388 (0.001)	0.028 (0.051)	0.105 (0.002)	0	0.016	0.021	0.245
Unweighted	0.077 (0.051)	0.06 (0.038)	0.007 (0.286)	0.137	0.08	0.033	0.037
**All**							
Weighted	0.995 (0.001)	0 (0.453)	0 (0.749)	0	0	0	0
Unweighted	1.042* (0.001)	0.001 (0.28)	0 (0.861)	0	0	0.001	0

Values in the table represent how many percent could the variables explain the PCC variation and values in parentheses represent the *P* value based on Monte Carlo permutation test. The asterisk (*) which the individual fractions of Env accounted more than 1 since there were negative R^2^_adj_ value during the redundancy analysis, negative R^2^_adj_ means that the explanatory variable can account for a smaller proportion of the total variation than that of the randomly generated variable. Therefore, usually in practical application, negative R^2^_adj_ is usually treated as 0, but it must be labeled as true R^2^_adj_ in order for all R^2^_adj_ components to add up to 1 (Referenced from Legendre and Legendre, 2012).

### Distinct seasonal features at the phylum and OTU levels

There were notable differences in the proportions of various phyla among different seasons (sampling month). In May, there was a greater proportion of Alphaproteobacteria (41.41%), Planctomycetes (6.42%), Actinobacteria (3.86%), Firmicutes (1.48%), Acidobacteria (0.45%), TM7 (0.16%), Tenericutes (0.16%), OD1 (0.13%), and WPS-2 (0.09%) than in Aug and Oct, whereas Gammaproteobacteria (44.23%), GN02 (0.08%) and SAR406 (0.04%) were depleted in May and Aug but enriched in Oct. In Aug, Bacteroidetes (13.98%), Deltaproteobacteria (6.93%), Verrucomicrobia (4.5%), Chloroflexi (0.36%), Lentisphaerae (0.97%), TM6 (0.25%), Nitrospirae (0.08%), Chlamydiae (0.07%), Chlorobi (0.07%), Spirochaetes (0.04%) and OP8 (0.03%) were significantly enriched than in the other two sampling times (Duncan test; Table S2).

**Figure 4:**
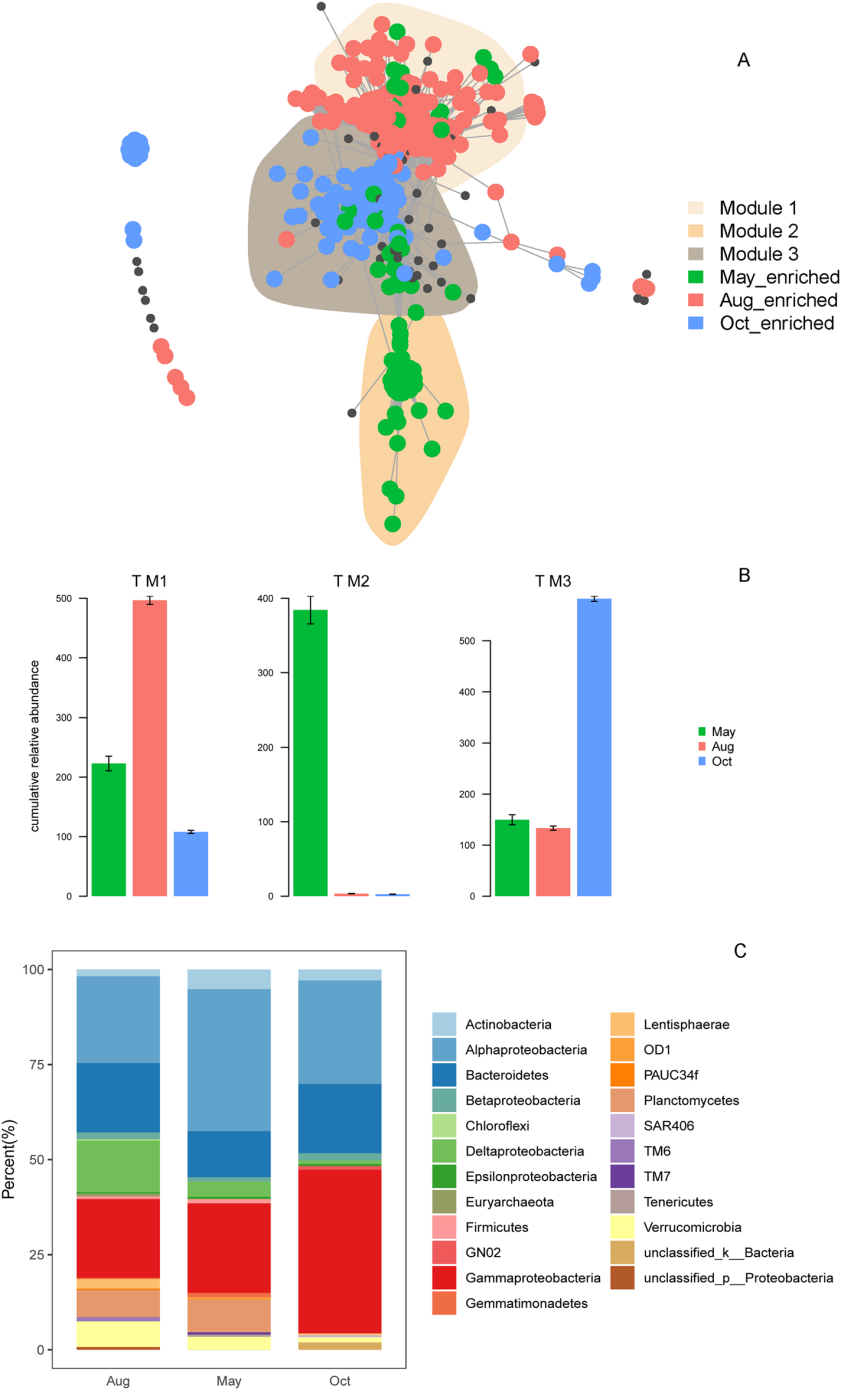
Co-occurrence patterns of seasonal sensitive OTUs (**A**). Co-occurrence network visualizing significant correlations (ρ > 0.7, *P* < 0.001; indicated with grey lines) between OTUs from the three sampling times with abundance > 0.01%. Different colors represent ssOTUs in May (green), Aug (red) and Oct (blue). Cumulative relative abundance (as counts per million, CPM; y-axis in × 1000) of all the sensitive modules in the networks (**B**). The phylum attribution of ssOTUs in each season (**C**). The y-axis is the percentage of the number of OTUs that belong to a particular phylum that accounts for the total number of all the OTUs.

At the OTU level, OTUs with relative abundance > 0.01% (1040 OTUs) were used to perform the difference analysis, and 175 OTUs in May, 281 OTUs in Aug, and 210 OTUs in Oct were identified as seasonal specific OTUs (ssOTUs). The cooccurrence network showed that the ssOTUs were clustered separately (Fig. 4A). Furthermore, the separation of the three modules contained most of the ssOTUs specific to different seasons (Fig. 4A-B). All the ssOTUs of different seasons comprised a taxonomically broad set of prokaryotes at the phylum (phylum Proteobacteria is grouped at the class level) level (Fig. 4C) belonging to various phyla with different proportions. Betaproteobacteria, Verrucomicrobia, Gemmatimonadetes, Epsilonproteobacteria, PAUC34f., and Euryarchaeota did not show significant differences among the three sampling times at the phylum level, but features belonging to these phyla showed differences at the OTU level (Fig. 4C, Dataset S4). In addition, the phylum ssOTUs belonging to, such as Alphaproteobacteria, Gammaproteobacteria, Bacteroidetes, Actinobacteria, and Deltaproteobacteria, were not only enriched at one sampling time (Dataset S4) but also enriched at the other two sampling times (Fig. 4C, Dataset S4). These results revealed that different seasons do not strictly select specific microbial lineages at the phylum level, but a finer level analysis could more strictly reflect the seasonal variation.

Regression analysis between the relative abundance of modules to which the ssOTUs belonged and the environmental factors was also conducted, and module 1 abundance, to which the Aug-ssOTUs belonged, showed a significant positive correlation with temperature (R^2^ = 0.77, *P* = 6.609e−62), AN (R^2^ = 0.43, *P* = 7.416e−25), and un_ionN (R^2^ = 0.75, *P* = 1.366e−58) and a negative correlation with SRP (R^2^ = 0.81, *P* = 6.762e-17). This may be caused by the functional role of the microbes in Aug. In the Aug-ssOTUs, Deltaproteobacteria showed a higher ratio than in the other 2 months (Fig. 4c), and in the following functional analysis, Deltaproteobacteria contributed to the genes related to nitrogen fixation, which may help to explain why there was a positive correlation of Aug-ssOTUs to AN and un_ionN. The module 2 abundance to which the May-ssOTUs belonged showed a significant negative correlation with pH (R^2^ = 0.65, *P* = 4.026e−44), temperature (R^2^ = 0.19, P = 2.325e−10), un_ionN (R^2^ = 0.025, *P* = 0.01779), and SRP (R^2^ = 0.12, *P* = 4.104e−07) and a positive correlation with AN (R^2^ = 0.26, *P* = 5.174e−14). In the May-ssOTUs, the ratio of Alphaproteobacteria was the highest, and Alphaproteobacteria were reported to be pH-sensitive groups in marine environments^20^, which prefer neutral pH environments^21^. In this study, the pH in May was 8.04 ± 0.07, in Aug was 8.39 ± 0.09, in Oct was 8.38 ± 0.07, and the pH in May was the closest to neutral, and the ratio decreased with increasing pH in Oct and Aug. The abundance of module 3, to which the Oct-ssOTUs belonged, showed a significant positive correlation with SRP (R^2^ = 0.81, *P* = 0.16e-10) and pH (R^2^ = 0.054, *P* = 0.00075) and a negative correlation with temperature (R^2^ = 0.44, *P* = 2.276e−25), AN (R^2^ = 0.75, *P* = 4.51e−58), and un_ionN (R^2^ = 0.6, *P* = 3.995e-39) (Fig. S2). Phosphate has been identified to limit primary productivity^22^, which is of great importance in the structure of dominant bacterial taxa in marine environments^23^. In the Oct-ssOTUs, the ratio of Gammaproteobacteria was the highest, as reported. Gammaproteobacteria was significantly explained by SRP during the seasonal variation in the Western English Channel, with Rho equal to 0.75^23^, which suggested the sensitivity of it to SRP, and in that study, it also showed a negative correlation between temperature and Gammaproteobacteria and a positive correlation between SRP and Gammaproteobacteria. Although the correlation was not significant, the variation trend was consistent, which indicates that the phenomenon observed in this study was not unexpected. In addition, most ammonia-oxidizing bacteria belong to the Betaproteobacteria and Gammaproteobacteria classes are chemolithoautotrophs that oxidize ammonia to nitrite^24^. Gammaproteobacteria and Betaproteobacteria both had higher ratios in Oct-ssOTUs, and the functional prediction results also showed that pmoA/amoA and pmoB/amoB, which encode ammonia monooxygenase, were mainly contributed by OTUs from Gammaproteobacteria and Betaproteobacteria (Dataset S10). The utilization of ammonia may explain the negative correlation between the Oct-ssOTUs and AN.

### Community assembly processes across different sampling months and sites

**Figure 5:**
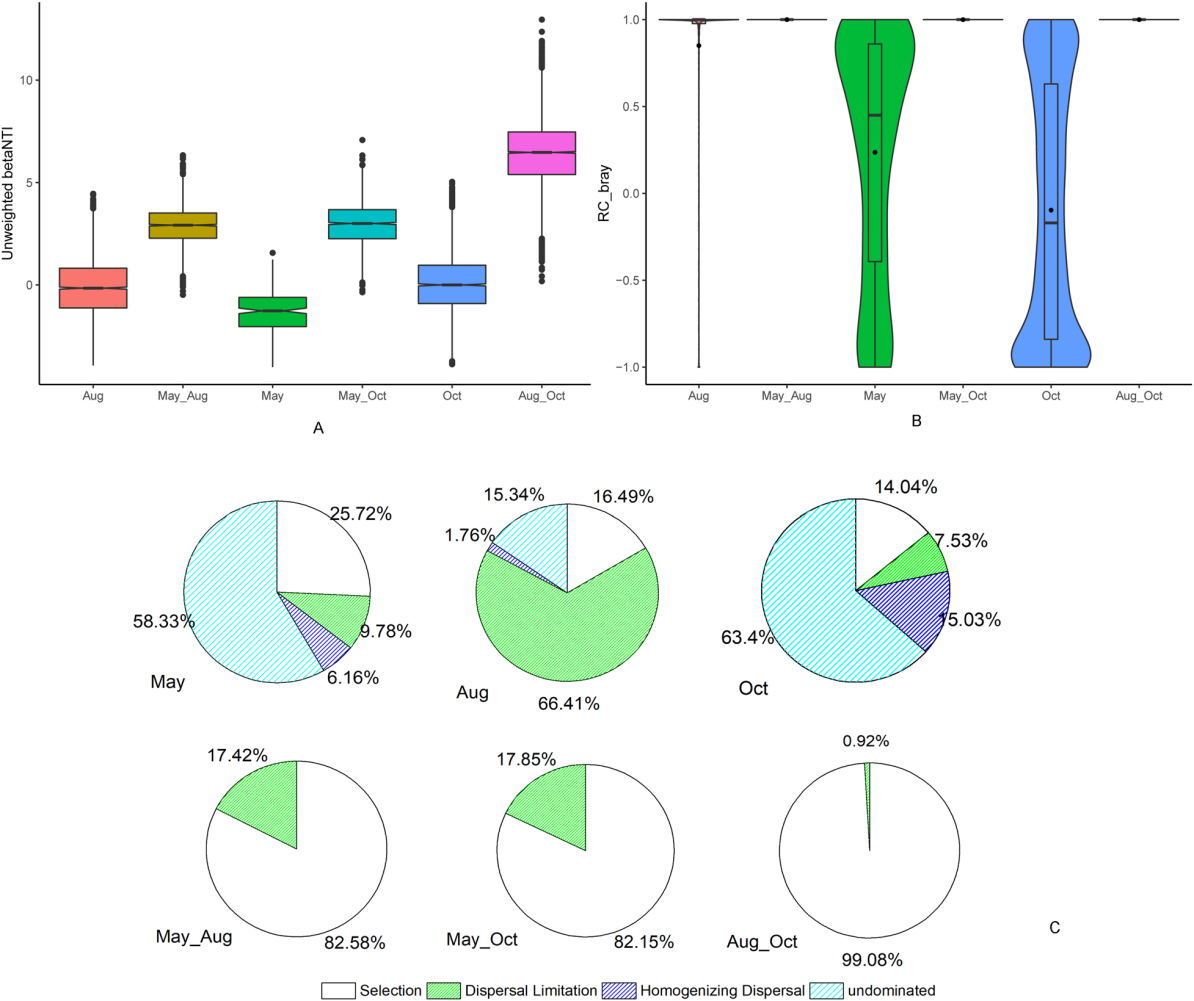
Patterns of distribution of unweighted *β*NTI (**A**) and RCbray (**B**) values across different sampling times. Quantification of the features that impose community assembly processes in and among different sampling times. (**C**) Pie charts give the percent of turnover in community composition governed primarily by Selection acting alone (white fill), Dispersal Limitation (green line fill), Homogenizing Dispersal (blue line fill) and undominated process (cyan fill).

Based on the analysis of phylogenetic turnover, unweighted *β*NTI mostly ranged from -2 to 2 across different sites at a single sampling time in May, Aug and Oct, revealing that PCC variations across different sampling sites at a single time were mostly affected by stochastic processes. The unweighted *β*NTI was greater than 2 across May–Aug, May–Oct and Aug-Oct (Fig. 5A), which revealed that the variations in PCC across different sampling times were mostly affected by deterministic processes. The RCbray values across any two sampling times were equal to 1, and in each sampling time, the RCbray values ranged from − 1 to 1 (Fig. 5B). Combining the *β*NTI and RCbray values, the community assembly processes were quantified at each sampling time and at any two sampling times. As shown in Fig. 5C, turning over of the community during different sampling times was mainly governed by selection; among the different sites in May and Oct, it was mainly governed by “undominated” processes; community turn over in Aug was mainly governed by the influence of “Dispersal Limitation”. These results indicated that the shifts in the assembly of prokaryotic communities during different sampling times were caused by strong “heterogeneous selection” (*β*NTI > 2), and the community variation at each sampling time was mainly caused by stochastic processes.

### Prediction of the metabolic potential at different sampling times

The NSTI scores of each sample ranged from 0.033 to 0.096, with a mean of 0.058 (Dataset S5). Microbial functions were detected in all the samples from the three sampling times, and it was found that the relative abundances of 242 pathways were significantly changed between samples from May and samples from Aug (Dataset S6). The relative abundances of 321 pathways were significantly changed between samples from May and Oct (Dataset S7), and the relative abundances of 370 pathways were significantly changed between samples from Aug and Oct (Dataset S8).

**Figure 6:**
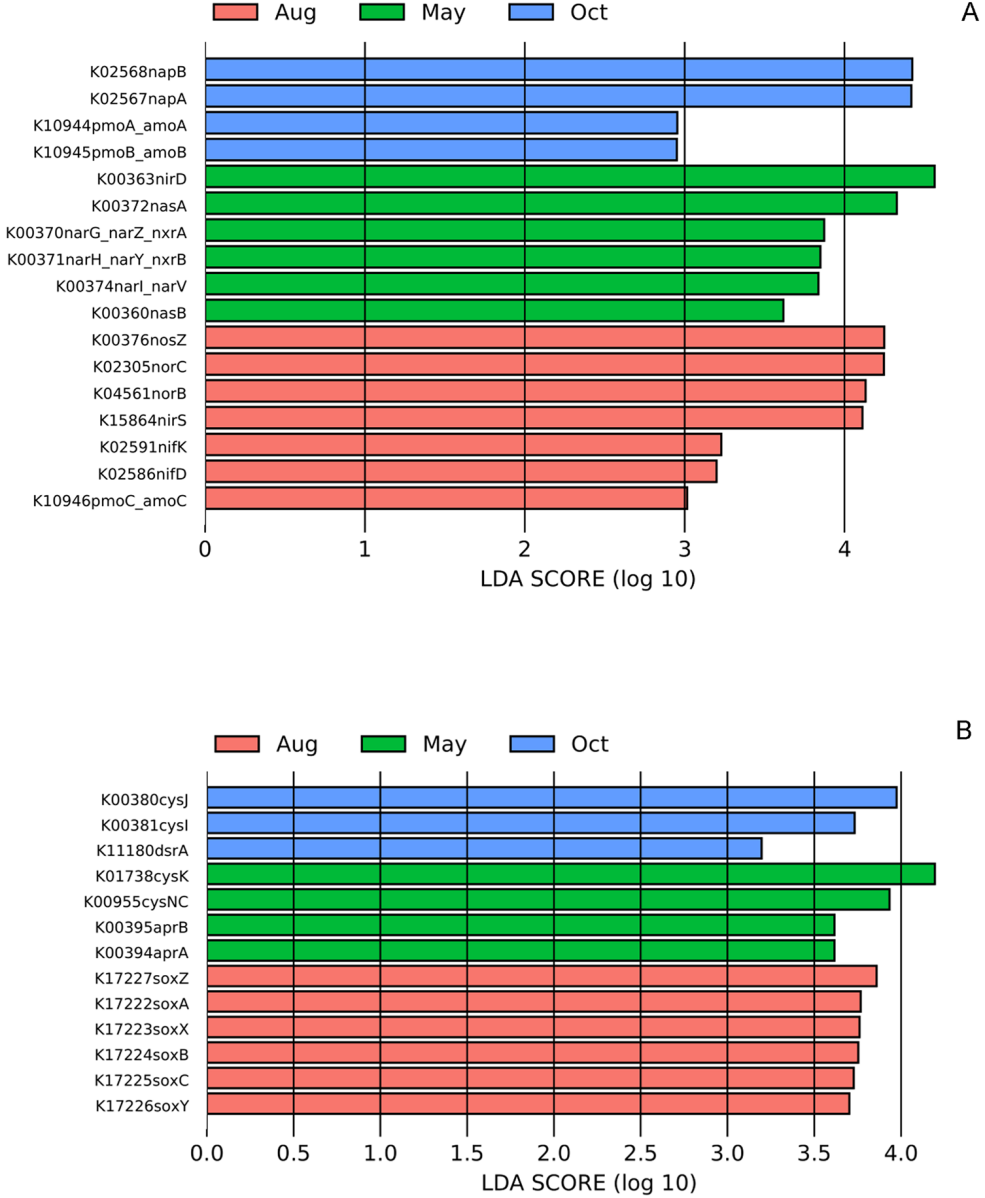
The LEfSe analysis indicated significantly differential abundances of PICRUSt predicted genes relevant to energy metabolism in different months of samples.

Genes related to energy metabolism were given more attention. For nitrogen metabolism genes relevant with nitrogen fixation (*nifD*, *nifK*) were detected only enriched in Aug, while genes relevant with nitrate reduction and denitrification (*narG*, *narZ*, *nxrA*, *narH*, *narY*, *nxrB*, *narI, narV*, *nirD*, *nasA*, *nasB*) were detected enriched in May, genes related with ammonia oxidation were both detected enriched in Oct and Aug. For sulfur metabolism, genes relevant with thiosulfate oxidation (*soxA, soxB, soxC, soxX, soxY and soxZ*) were only enriched in Aug, while genes relevant with sulfate and sulfite reduction (*cysNC*, *aprA*, *aprB*, *cysJ*, *cysI*, *cysK, dsrA*) were detected enriched in May and Oct (Fig. 6).

Prokaryotic taxa contributed to the significantly varied functional genes related to nitrogen and sulfur metabolism at different sampling times. At the species level, the taxa contributing to *nifK* and *nifD* mainly belonged to Deltaproteobacteria and Firmicutes, and the taxa contributing to the sox-series genes mainly belonged to Alphaproteobacteria and Gammaproteobacteria (Fig. S3)*.* The denitrification-related functional genes that were enriched in May were mainly contributed by members from Alphaproteobacteria and Gammaproteobacteria. The taxa contributing to *dsrA*, *aprA* and *aprB* were mainly from Deltaproteobacteria, including members of Desulfarculaceae, Desulfobacteraceae, Desulfobulbaceae, Desulfovibrionaceae and Syntrophobacteraceae (Fig. S4).

## Discussion

We found clear temporal variation in the composition of the prokaryotic community in the surface water of Bohai Bay across different sites during the three sampling months. We observed that environmental factors have a stronger effect on PCC variation than spatial factors during different sampling times. Overall, environmental factors such as temperature, pH, AN, un_ionN and SRP all showed significant correlations with α/*β*-diversity (Dataset S2, Table 2). In addition, the coefficients between environmental factors (temperature, pH, AN, un_ionN, COD, SRP, DO, DIN) and *β*-diversity/*β*NTI were decreased when the temporal factor was deconvoluted (Tables 2, 4), which suggested that these factors were also seasonally varied, and it can be speculated that seasonally varied environmental factors contributed to the dramatic prokaryotic temporal variation. Temperature plays a fundamental role in regulating the activity and growth of microorganisms, and changes in temperature could have a major direct effect on the population dynamics of marine bacteria^25^. In this study, temperature had the highest correlation coefficient with unweighted *β*-diversity (Table 2), which indicated its important role in affecting seasonal community variation. pH is also an important factor in shaping marine microbial communities^20,26,27^. Many studies have focused on the influence of pH decrease as a consequence of ocean acidification on marine zooplankton^26,28,29^. Acidification may impact prokaryotic communities by shifting towards plankton dominance, affecting productivity and nutrient cycling as well as increasing the abundance of disease-causing bacteria^20,28^. In contrast to previous studies, the community shift with pH variation in this study seemed not to be caused by ocean acidification, since the previously reported increase in the abundance of Flavobacteriales (Bacteroidetes)^20^ and disease-causing bacteria, such as Vibrionaceae and Alteromonadaceae (Gammaproteobacteria)^26,28^, at lower pH values was not observed in Bohai Bay in May (Table S2). Conversely, the pH observed in this study was slightly alkaline, with which in Aug was the highest. The rising temperature and strong photosynthesis in Aug may reduce the concentration of carbon dioxide and hydrogen ions in the surface seawater, resulting in a rise in pH. In addition, the riverine input and anthropogenic activities during different seasons may also affect the pH value. AN was reported to be an important factor in the marine environment affecting the variation in OTU richness^30^. In a previous study of the same research scope in Bohai Bay ^18^, AN showed a significant correlation with the *β*-diversity of the bacterioplankton community along with differently polluted areas. In this study, there was a positive correlation between AN and OTU richness and phylogenetic diversity (faith_pd) (Dataset S2), and the correlation coefficient between AN and the weighted UniFrac distance-based *β*-diversity was the highest (Table 2). The correlation between un_ionN and *α/β*-diversity was consistent with that of AN, and even with higher coefficients (Tables 2 & 4, Dataset S2), the effect of un_ionN on the microbial community was rarely reported, since un_ionN is a part of AN, and in this study, un_ionN exhibited a different influence on the distribution of ssOTUs among seasons (Fig. S2) with AN, which may indicate that the different chemical forms on phylogenetic distribution are different, and more targeted research is needed to explain this phenomenon. There was a negative correlation between SRP and the prokaryotic *α*-diversity in all samples, while the analysis based on a single sampling time showed that it was in August that the *α*-diversity indexes (faith_pd, richness, Shannon, and Pielou_e) were all negatively correlated with SRP (Dataset S2), which indicated that it may be the heterogeneous input of phosphate in different stations in August that caused the variation. Gilbert et al.^31^ reported that the most abundant phyla varied among different seasons in the Western English Channel and were associated with SRP. Here, we also found the importance of SRP in shaping the phylogenetic structure of the prokaryotic community. Phosphate has been identified to limit primary productivity ^22^, and in this study, phosphate played an important role in structuring the dominant taxa enriched in Oct (ssOTUs in Oct), which was mainly composed of members from Gammaproteobacteria (Fig. 4c), whose variation was also reported to be significantly explained by the SRP variable in the marine environment^23^. COD was also found to be an important factor significantly correlated with *β*-diversity and *β*NTI (Tables 2, 3) in this study. COD serves as one of the comprehensive indexes of the relative content of organic matter, which reflects the degree of pollution by reducing substances in the water. In the coastal region of the East China Sea, it was also found that COD had a significant effect on the microbial community composition^4^. The COD with the highest value was observed in Oct in Bohai Bay in this study, which may be caused by the apoptosis of phytoplankton since *Chl_a* was the lowest in Oct, which may contribute to the higher COD. In addition, the seasonal COD flux and tidal influence may also affect the transport processes of COD in the Bohai Bay area in different seasons^32^. DO and DIN had relatively lower but significant correlations with *α/β*-diversity and *β*NTI than other parameters, but they were both reported as very important factors in shaping marine bacterial communities^18,33–35^. In conclusion, the coexistence of multiple seasonally varied environmental factors shows a significant association with the prokaryotic seasonal pattern of surface water in the study area.

**Table 4:** Spearman’s rank correlation between environmental/spatial variability (Euclidean distance) and weighted and unweighted *β*NTI among all samples from different season.

Variables	Weighted					Unweighted				
	**r**	**p**	**r_**_**temporal**_	**p_**_**temporal**_	**N**	**r**	**p**	**r_**_**temporal**_	**p_**_**temporal**_	**N**
pH	− 0.1049	0.975	− 0.3321	1	68	0.02956	0.142	− 0.6056	1	68
AN	**0.3439**	0.001	**0.1858**	0.001	68	**0.5913**	0.001	**0.2918**	0.001	68
Env	**0.1673**	0.007	0.04493	0.231	68	**0.3306**	0.001	0.03383	0.135	68
Temp	**0.3442**	0.001	**0.2275**	0.001	68	**0.6558**	0.001	**0.4364**	0.001	68
un_ionN	**0.4053**	0.001	**0.3115**	0.001	68	**0.7201**	0.001	**0.6174**	0.001	68
COD	**0.2293**	0.001	**0.1245**	0.002	68	**0.3853**	0.001	**0.1345**	0.002	68
Nitrite	0.08334	0.05	0.07562	0.125	68	0.003717	0.385	− 0.0406	0.888	68
SRP	**0.2698**	0.001	**0.1957**	0.002	68	**0.3919**	0.001	**0.321**	0.001	68
Salinity	− 0.02396	0.671	− 0.1029	0.988	68	0.02563	0.123	− 0.1433	1	68
DO	**0.102**	0.042	0.006755	0.441	68	**0.2327**	0.001	**0.123**	0.001	68
DIN	**0.09197**	0.038	0.01272	0.4	68	**0.1929**	0.001	**0.08925**	0.005	68
Chlorophyll_α	− 0.1087	0.985	− 0.1467	0.998	68	− 0.003634	0.54	− 0.202	1	68
Nitrate_N	− 0.003856	0.555	− 0.03669	0.716	68	0.009555	0.35	− 0.04097	0.926	68
Depth	0.03468	0.245	0.017	0.327	68	− 0.0894	1	− 0.1126	1	68
Trans	− 0.02311	0.708	− 0.03759	0.747	68	− 0.007616	0.608	− 0.08974	1	68
Geo	0.04925	0.151	0.04158	0.241	68	− 0.05309	0.98	− 0.07174	0.989	68
Longitude	0.03519	0.265	0.03333	0.284	68	− 0.04764	0.97	− 0.06673	0.981	68
Latitude	0.00531	0.397	0.02266	0.28	68	− 0.01947	0.819	− 0.01521	0.721	68
Conductivity	− 0.09627	0.969	− 0.1104	0.968	68	0.03493	0.105	0.02299	0.191	68

r, correlation coefficients between environmental/spatial variability and prokaryotic community *β*NTI derived from Mantel testes with 999 permutations, r__temporal_, correlation coefficients between environmental/spatial variability and prokaryotic community *β*NTI controlled by temporal variation derived from partial Mantel testes with 999 permutations. Data in bold indicate significant correlations (*P* < 0.05). Refer to Table 1 and Table 2 for abbreviations.

According to the quantitative analysis of assembly processes among different months, it could be clearly seen that the selection processes accounted for most (> 80%) of the turnover of the community between any two sampling times, and combined with the *β*NTI value mostly above 2, it can be speculated that the “heterogeneous selection” processes dominated the temporal community variation (Fig. 5). The temporal effect was so strong that the spatial effect was masked by it during different sampling times. Recently, increasing evidence has shown that the spatial distributions of microbes are nonrandom and that distance decay of similarity occurs for microbial compositional structures^36,37^. Similar findings have been made in Bohai Bay as a result of our previous research within the study area, similar to this work^18^. To eliminate the influence of seasonal factors on spatial effects, we also tested the environmental-spatial effect on PCC variation for single season/month samples. According to the VPA results, no factors could explain the community variation in May (Table 3), and the assembly process quantification results showed that the “undominated” processes accounted for 58.33% of the community turnover in May among the 12 sites, while the “selection process” accounted for 25.72%, which indicated that the communities in May were mostly governed by stochastic processes, and there may be unknown environmental factors that were not detected that accounted for the “selection process” parts (Fig. 5C). In Aug, the environmental and spatial factors did not show purely significant effects on the PCC variation, while their joint effects could explain 49.5% of the PCC variation (based on UUF distance) among the 28 sites in this sampling time (Table 3), and the dispersal limitation accounted for 66.41% of the community turnover in Aug (Fig. 5C), which suggested that spatial factors play an important role in determining the PCC variation in Aug, since in multivariate analysis, when using variation partitioning^38^ to estimate the relative importance of environmental and spatial factors, the spatial factors often considered to infer the dispersal process in microbial community assembly in marine waters, which could be considered as spatial effects. In October, the VPA results based on unweighted UniFrac distances showed that the environmental and spatial factors could explain 34.7% of the PCC variation. Meanwhile, the quantitative analysis of assembly processes based on unweighted *β*NTI showed that the selection and dispersal process accounted for 36.6% of the community turnover, which was consistent with the VPA results (Table 3, Fig. 5C). From the above, we could conclude that environmental and spatial factors driving the assembly processes on the PCC variation were changed with the temporal shifts.

The detection of dominant groups within microbial assemblages may be of particular importance in ecological studies concerning biogeochemical cycles^2^, as microbes belonging to different groups can express different degrees of activity in a given ecosystem^39^. The groups of microorganisms that varied with the season variation were different in different studies. For example, Gammaproteobacteria in the SPOT observation was the highest in August and the lowest in May, Flavobacteria was highest in February and lowest in Oct, while Cyanobacteria was highest in November and lowest in April^6^. A similar study on the Qinhuangdao coast of the Bohai Sea found that Alphaproteobacteria and Firmicutes were the most abundant in July, while Cyanobacteria and Bacteroidetes were the most abundant in Oct^5^. In contrast, in this study, the abundance of Gammaproteobacteria was highest in Oct and lowest in May, that of Alphaproteobacteria and Firmicutes was highest in May, while that of Bacteroidetes was highest in August. In contrast, Cyanobacteria did not show significant differences at each sampling time during the three sampling times in different seasons (Table S2). The differences between studies in different regions indicate that seasonal variation in microorganisms is not consistent across the region, even in different regions of the same ocean. In addition to differences in detection methods (qPCR/ARISA/Illumina MiSeq), the differences in the results further indicate that except for seasonal factors, other local environmental factors may also play an important role in determining prokaryotic community patterns. Further study is needed to focus on seasonal changes in microbial communities in different marine environments.

This study paid more attention to OTUs that varied dramatically among the detected months with the aim of finding their correlation with the local environmental factors, which may provide some clues for the prediction of how the microbial community reacts to changes in the marine bay environment. We found that the ssOTUs naturally clustered as different modules in the network analysis (Fig. 4A), and the cumulative relative abundance of the ssOTUs in each module all showed definite seasonal bias (Fig. 4B). Moreover, the ssOTUs had a significant correlation with the environmental factors (Fig. S2, Temperature, AN, un_ionN, SRP, pH), which was consistent with the driving factors related to the PCC temporal variation obtained from the Mantel test based on *β*-diversity. These results indicated that the seasonally varied environmental factors definitely affected the variation in prokaryotic communities at the OTU level. Many studies have aimed to identify the specific taxonomic groups that drive seasonal variation, and it was found that nonseasonal taxa contain seasonal OTUs^6^, which calls for finer taxonomic groups to be analysed since they may adapt to different ecological niches using different metabolic genes. In this study, the analysis of ssOTUs could reflect the response of the prokaryotic communities to temporal environmental changes more elaborately and comprehensively. It was found that some ssOTUs within the phyla to which they belonged (Verrucomicrobia, Betaproteobacteria, Crenarchaeota) did not show significant temporal variation, and most of the ssOTUs in different seasons came from the same phylum. This suggested that it may not be enough to analyse the community at only the phylum level but may be necessary to pay more attention to finer levels.

Temporal functional gene variation has rarely been studied in seasonal research on marine environments, such as in Bohai Bay. In this study, PICRUSt2 was used to predict the approximate functional potential of the prokaryotic community. The accuracy of PICRUSt generally increased with decreasing NSTI score, and reliable results were generated from a dataset of soil samples with a mean NSTI score of 0.17. In this study, the mean NSTI score was 0.058, suggesting that the results are credible and could accurately reflect the variation trend of the targeted function. Based on the PICRUSt2 results, the nitrogen fixation and sulfur oxidation functions were mainly enriched in August, while the denitrification and sulfite or sulfate reduction functions were mainly enriched in May and October (Fig. 6). The variation in nitrogen metabolism during different seasons in this study was consistent with a study of oceanic mangrove habitats, where the rate of nitrogen fixation in the wet season was higher than that in the dry season, while the potential denitrification rates were higher during the dry season than during the wet season^40^. The variation in sulfur metabolism in different seasons in surface seawater has rarely been reported, since it is often reported to occur in anaerobic environments, and most studies are concerned with the variation in sulfur metabolism in lakes and marine sediments^16,41^. In this study, the nitrogen fixation and sulfur oxidation functions were predicted to be enriched in August, which may be because rainfall in the wet season (Fig. S6) is often accompanied by periodic high tides, which bring abundant dissolved nutrients, such as SRP, nitrate, and ammonia nitrogen, which could effectively improve the availability of nitrogen or sulfur, promoting the enrichment of nitrogen fixation and sulfur oxidation. However, the anaerobic functions mediated by sulfate-reducing bacteria and denitrifying bacteria that were enriched in May and Oct should be carefully interpreted. This phenomenon may be explained by the relative abundance of the prokaryotic taxa that contributed to the denitrification and sulfate reduction functions. Prokaryotic taxa contributing to these two functions at the species level showed a very low relative abundance of minus than 0.01 for most of them (Fig. S5), except for taxa belonging to Rhodobacteraceae, which contained members with aerobic denitrification abilities, such as *Paracoccus*, a representative aerobic denitrifying bacterium that was commonly found to be dominant in sulfate reduction bacteria (SBR) during the denitrification process^42^. The contributed taxa varied among the three sampling times, resulting in the corresponding functional variation, while this was not to say that the detected function played a key role in the corresponding period; it could just reflect the relative change in a certain function during the sampling period in different seasons. However, the distinct variation in nitrogen and sulfur metabolism-related genes distributed in different sampling months reminds us to pay more attention to the nitrogen and sulfur cycles in surface seawaters in future research.

The linkage between nitrogen and sulfur metabolism is also worth attention. In this study, both the denitrification and sulfate reduction potential functions were predicted to be enriched in May and Oct, which may indicate that these two processes are interwoven with each other in the surface water of Bohai Bay. These predicted denitrifying members of Alphaproteobacteria and Gammaproteobacteria were most negatively correlated with nitrogen nutrients (Dataset S9), which may suggest the strengthened consumption of nitrogen salts by these taxa in October and May. Simultaneously, the sulfite and sulfate reduction members of Deltaproteobacteria were most positively correlated with un_ionN, which may indicate the relevance between sulfate reduction and nitrogen metabolism. These findings may guide further study of nitrogen and sulfur metabolism in Bohai Bay, and more reliable analytical techniques, such as metagenomic and meta-transcriptomic sequencing, will be needed in future work.

Finally, due to limited sampling, samples from the cold season were not collected, while the aim of this study was to explore the spatial and temporal effects on the distribution of prokaryotic communities. The results clearly showed strong “heterogeneous selection” processes affecting the PCC temporal variation during the study period. The VPA and quantitative analysis of the assembly processes showed that environmental and spatial factors driving the assembly processes on the PCC variation were changed with the temporal shifts. There were distinct seasonal features at the phylum and OTU levels (ssOTUs), while the ssOTUs exhibited different seasonal attributions under the same phylum, which calls for finer-level analysis to reflect the temporal variation more strictly. Moreover, functional prediction showed that nitrogen and sulfur metabolism were significantly shifted during the temporal variation, which needs further study for nitrogen and sulfur metabolism in Bohai Bay.

## Materials and methods

### Study area, sampling, and physicochemical analyses of seawater samples

We collected 188 (biological replicates included) surface seawater samples from 12 to 28 stations (Fig. S1), which covered the north and south coasts and the offshore area of Bohai Bay. Among the sampling stations, TJ19 was located nearest the Bei Pai River mouth, TJ12 was located near the Du Liu Jian River in the southern part, TJ04 was located near the Yong Ding Xin River in the northern part, TJ01 was located near Da Sheng Tang village, TJ02 was located near Ma Zu park, TJ21 was located near Dongjiang Bay, which are all popular tourist attractions in the Tianjin coastal region, TJ13, TJ09, TJ20, TJ24 and TJ30 were located at a middle distance off the shore, and the most remote station, TJ14, was located as far as 110 km off the shore, which was more influenced by ocean currents from the Bohai Sea than other stations. Surface water samples at 0.5 m depth were collected at each site during 16–18 May, 16–18 August (Aug) and 17–19 October (Oct) in 2016. In May, samples from 12 stations were collected, and in August and October, samples from 28 stations were collected. In Aug and Oct, each site was sampled with three replicates and in May with two replicates. The detailed sampling information is listed in Dataset S1. All sampling was undertaken during high tide conditions. Approximately 500 ml of the water sample of each sample was filtered onto a 0.2 μm polycarbonate membrane (Millipore, USA) and stored at − 20 °C prior to DNA extraction. Environmental DNA on the polycarbonate membrane was extracted using a PowerWater DNA Isolation Kit (MO BIO, USA) according to the manufacturer’s instructions. Sampling environmental parameters, including water temperature, depth and transparency, were recorded through field measurements. The pH values, DO, salinity and conductivity were measured using a probe (Multi 3430, WTW, Germany). The concentrations of soluble reactive phosphate (SRP), ammonium nitrogen (AN), nitrite, nitrate, dissolved inorganic nitrogen (DIN) and COD were analysed following the standard methods of the specification for marine monitoring of seawater analysis (GB 17378.4-2007). Chlorophyll a (*Chl*_a) was determined in water samples in accordance with the “Specification for oceanographic survey” (GB/T 12763.4-2007). Nonionic ammonia (un_ionN) was determined in accordance with the “Surface water quality standards” (GB 3838-88). The geographic coordinates and physicochemical parameters of the water samples are listed in Dataset S1. Student’s t test was used to compare the environmental difference between any two time points. Principal component analysis (PCA) was performed to reveal the relationships between the environmental parameters and the distribution of the sampling stations and time in an ordination plot.

### DNA extraction, 16S rRNA gene amplification, and Illumina sequencing

The DNA extracts were quantified using a spectrophotometer (NanoDrop ND-2000, USA) and subsequently submitted to Novogene Co. Beijing for 16S rRNA gene amplification and sequencing. Briefly, the 16S rRNA gene V4 region was amplified using dual-indexed bacterial/archaeal primers 515 f. (GTG YCA GCM GCC GCG GTA A) and 806r (GGA CTA CNV GGG TWT CTA AT) containing adaptor sequences for the Illumina HiSeq 2500 platform (Illumina, San Diego, CA, USA) using the 2 × 250PE rapid run mode. Details of the sequencing libraries can be found in previous work^18^.

### 16S rRNA gene amplicon processing and analysis

Sequences were demultiplexed in Quantitative Insights Into Microbial Ecology version 2 (QIIME2). The raw sequences with forwards and reverse reads were imported through *time QIIME tools import* with PairedEndFastqManifestPhred33V2 as input format, and amplicon sequence variants were generated using DADA2 version 2019.7.0 with *time QIIME dada2 denoise-paired* script to remove sequences with quality scores < 20 and chimeric sequences^43^. FeatureData[Sequence] were obtained after quality control with DADA2, which is the equivalent of the QIIME 1 representative sequences file, and which are the equivalent of 100% OTUs from QIIME 1 with more accurate estimates of diversity and taxonomic composition, which are generally referred to as amplicon sequence variants (ASVs). The phylogenetic tree was generated based on the representative sequences using align-to-tree-mafft-fasttree from the Q2-Phylogeny plugin. Taxonomy was assigned using the q2-feature-classifier command with default parameters in QIIME2, and sequences were matched against the Greengenes 13_8 database^44^.

Prokaryotic *α-*diversity and *β*-diversity estimates were calculated by even rarefication at 26,000 reads per sample, with multiple indexes (Faith Phylogenetic Diversity metric (Faith_pd), the quantification of taxon richness through phylogenetic tree units (OTU_richness), Shannon and evenness (pielou_e) and weighted (WUF)/unweighted UniFrac (UUF) distances between samples. The differences in *α-*diversity indexes and taxa abundances at the phylum level among different sampling times were analysed by one-way ANOVA with alpha levels obtained by a two-tailed test. Pearson’s correlation coefficients between geo-physical–chemical variables and *α-*diversity indexes were calculated to measure the relationships between them. We used a principal coordinates analysis (PCoA) plot based on WUF and UUF distances to visualize the sample clusters and dissimilarity in prokaryotic community composition between sampling times and subsequently tested the significance of pairwise dissimilarity in community composition between sampling times with analysis of similarity (ANOSIM) and permutational multivariate analysis of variance (PERMANOVA). Simple and partial Mantel tests were used to test the correlations of geographic distance and environmental parameters with prokaryotic *β*-diversity (variation in community composition). Variance partitioning analysis (VPA) was conducted on all samples or single-time sampling using the WUF/UUF distances as response variables and using environmental factors, linear trends, and principle coordinates of neighbor matrices (PCNM) as the explanatory variables to investigate the relative contributions of spatial and environmental parameters to the variation in PCC, with the same analysis methods as those used in a former study in Bohai Bay^18^. The spatial parameters included linear trends and PCNM, and the environmental factors included all the physical–chemical parameters detected in Bohai Bay seawater samples.

The OTUs responsible for the differences among the sampling times were identified by correlation-based indicator species analysis using the function multipatt () in the package “indicspecies”^45^ and likelihood ratio tests using the function DGEList () in the package “edgeR”^46^. Finally, the OTUs confirmed by both approaches were defined as seasonal sensitive OTUs (ssOTUs). The cooccurrence network was performed on the OTUs with a relative abundance of > 0.01%. Then, the abundances of screened OTUs were normalized by the “trimmed means of M” (TMM) method and expressed as relative abundance per counts million (CPM) using the function cpm () in the package “edgeR”. The TMM-normalized CPM counts and calculated Spearman rank correlations (ρ > 0.7, *p* < 0.001) between OTUs were utilized to construct a meta co-occurrence network. To further investigate the relationship of ssOTUs and the community structure of different sampling times, we identified net-work modules, which are substructures of nodes with a higher density of edges within groups than between them. For this, we utilized the greedy optimization of modularity algorithm^47^ as implemented in the package “igraph”. Modules with the ssOTUs were further analysed to calculate the cumulative relative abundance using the TMM method; simultaneously, the regression analysis between the module abundance and environmental factors was also analysed through R^48^.

The functional potential of the prokaryotic community was predicted by PICRUSt2 (v2.1.4 beta)^49^ according to the guidelines with the unrarefied OTU abundance table as the input. The relative predicted abundance of KEGG pathways was calculated by dividing the abundance of each pathway by the sum of all pathway abundances per sample. The relative contribution of each OTU to the predicted pathways was calculated by dividing the contribution of each OTU by the sum of all contributions per sample. The significantly discriminant energy metabolism gene ontologies related to nitrogen and sulfur metabolism in each sampling time were determined using LDA effect size^50^, which employs the factorial Kruskal–Wallis sum-rank test (α = 0.05) to identify genes with significantly different relative abundances between sampling times (using one-against-all comparisons). The taxonomy assignment of the predicted genes associated with nitrogen or sulfur metabolism with relative abundance significantly different in each season was plotted using Heml 1.0^51^.

### Analysis of assembly processes of prokaryotic communities

The *β*-nearest taxon index (*β*NTI)^12^ and Bray–Curtis-based Raup–Crick (RCbray)^52^ were used to quantify community assembly processes and identify features that impose them. Based on the random shuffling of OTU labels across the tips of the phylogeny, the *β*NTI was computed as the number of standard deviations that observed *β*-mean nearest taxon distance (*β*MNTD) values deviated from the mean of the null distribution (999 null replicates)^12^. If the observed *β*MNTD is significantly greater or less than the null expected phylogenetic turnover (*β*NTI >  + 2 or <  − 2), we can infer that “heterogeneous selection” or “homogeneous selection” (under heterogeneous or homogeneous environmental conditions) governs phylogenetic difference or similarity between communities, respectively^53,54^. If |*β*NTI|< 2, stochastic processes may play an important role in structuring microbial communities^12,17^. The second step of the quantification was to use the modified Raup-Crick metric^55^ based on Bray‒Curtis dissimilarity to evaluate the standardized deviation of observed OTU compositional turnover from the null expectation^52^. When |*β*NTI|< 2, if the observed Bray‒Curtis dissimilarity is significantly greater or less than the null expected compositional turnover (RCbray >  + 0.95 or <  − 0.95), we can infer that “dispersal limitation” or “homogenizing dispersal” governs compositional difference or similarity between communities, respectively. RCbray values between − 0.95 and + 0.95 infer that the compositional turnover between a given pair of communities is “undominated” by any single process^54^. Subsequently, we calculated the percentages of each process in all pairwise comparisons according to the *β*NTI and RCbray values.

The boxplot or bar plot in Figs. 2, 3, 5 and 6 were made through the ImageGP web server^39^.

## Supplementary Information

Supplementary Information 1.

Supplementary Information 2.

## Data Availability

The raw data of sequencing samples have been deposited under the sequence read archive (SRA) section of NCBI under the following accession number: PRJNA645045, which are available from the SRA Run Selector: https://www.ncbi.nlm.nih.gov/Traces/study/?acc=PRJNA645045&o=acc_s%3Aa.

## References

[1] Celussi M, Bussani A, Cataletto B, Del NP (2011). Assemblages' structure and activity of bacterioplankton in northern Adriatic Sea surface waters: A 3-year case study. FEMS Microbiol. Ecol..

[2] Castle D, Kirchman DL (2004). Composition of estuarine bacterial communities assessed by denaturing gradient gel electrophoresis and fluorescence in situ hybridization. Limnol. Oceanogr. Methods.

[3] Fuhrman JA (2006). Annually reoccurring bacterial communities are predictable from ocean conditions. Proc. Natl. Acad. Sci. U.S.A..

[4] Wang K (2015). Bacterial biogeography in the coastal waters of northern Zhejiang, East China Sea is highly controlled by spatially structured environmental gradients. Environ. Microbiol..

[5] He YD (2017). Distinct seasonal patterns of bacterioplankton abundance and dominance of phyla alpha-proteobacteria and cyanobacteria in Qinhuangdao coastal waters off the Bohai Sea. Front. Microbiol..

[6] Cram JA (2015). Seasonal and interannual variability of the marine bacterioplankton community throughout the water column over ten years. ISME J..

[7] Xiong JB (2014). Biogeography of the sediment bacterial community responds to a nitrogen pollution gradient in the East China Sea. Appl. Environ. Microbiol..

[8] Du JK (2013). Temporal and spatial diversity of bacterial communities in coastal waters of the South China Sea. PLoS ONE.

[9] Suh SS (2015). Seasonal dynamics of marine microbial community in the South Sea of Korea. PLoS ONE.

[10] Zhang R, Lau SC, Ki JS, Thiyagarajan V, Qian PY (2009). Response of bacterioplankton community structures to hydrological conditions and anthropogenic pollution in contrasting subtropical environments. FEMS Microbiol. Ecol..

[11] Reza MS (2018). Basin-scale seasonal changes in marine free-living bacterioplankton community in the Ofunato Bay. Gene.

[12] Stegen JC, Lin X, Konopka AE, Fredrickson JK (2012). Stochastic and deterministic assembly processes in subsurface microbial communities. ISME J..

[13] Dini-Andreote F, Stegen JC, van Elsas JD, Salles JF (2015). Disentangling mechanisms that mediate the balance between stochastic and deterministic processes in microbial succession. Proc. Natl. Acad. Sci. U.S.A..

[14] Vellend M (2010). Conceptual synthesis in community ecology. Q. Rev. Biol..

[15] Chase JM, Myers JA (2011). Disentangling the importance of ecological niches from stochastic processes across scales. Philos. Trans. R. Soc. Lond. Ser. B Biol. Sci..

[16] Wang K (2016). Regional variations in the diversity and predicted metabolic potential of benthic prokaryotes in coastal northern Zhejiang, East China Sea. Sci. Rep..

[17] Zhang HJ (2018). Microbial community dynamics and assembly follow trajectories of an early-spring diatom bloom in a semienclosed bay. Appl. Environ. Microbiol..

[18] Zhao W (2020). Bacterioplankton community variation in Bohai Bay (China) is explained by joint effects of environmental and spatial factors. Microbiologyopen.

[19] Guo L, Sun M, Wang X, Lan H (2019). Statistical analysis of the characteristics difference of rainfall over Bohai Bay and the land. Clim. Change Res. Lett..

[20] Witt V, Wild C, Anthony KR, Diaz-Pulido G, Uthicke S (2011). Effects of ocean acidification on microbial community composition of, and oxygen fluxes through, biofilms from the Great Barrier Reef. Environ. Microbiol.

[21] Shore A, Day RD, Stewart JA, Burge CA (2021). Dichotomy between regulation of coral bacterial communities and calcification physiology under ocean acidification conditions. Appl. Environ. Microbiol..

[22] Krom M, Kress N, Gordon S (1991). Phosphorus limitation of primary productivity in the eastern Mediterranean Sea. Limnol. Oceanogr..

[23] Gilbert JA (2009). The seasonal structure of microbial communities in the Western English Channel. Environ. Microbiol..

[24] Arp DJ, Stein LY (2003). Metabolism of inorganic N compounds by ammonia-oxidizing bacteria. Crit Rev Biochem Mol Biol..

[25] Apple JK, Giorgio PAD, Kemp WM (2006). Temperature regulation of bacterial production, respiration, and growth efficiency in a temperate salt-marsh estuary. Aquat. Microb. Ecol..

[26] Fonseca F, Cerqueira R, Fuentes J (2019). Impact of ocean acidification on the intestinal microbiota of the marine Sea Bream (*Sparus aurata* L.). Front. Physiol..

[27] Lindh MV (2013). Consequences of increased temperature and acidification on bacterioplankton community composition during a mesocosm spring bloom in the Baltic Sea. Environ. Microbiol. Rep..

[28] Meron D (2011). The impact of reduced pH on the microbial community of the coral Acropora eurystoma. ISME J..

[29] Hassenruck C, Hofmann LC, Bischof K, Ramette A (2015). Seagrass biofilm communities at a naturally CO_2_-rich vent. Environ. Microbiol. Rep..

[30] Richa K (2017). Distribution, community composition, and potential metabolic activity of bacterioplankton in an urbanized Mediterranean Sea coastal zone. Appl. Environ. Microbiol..

[31] Gilbert JA, Field D, Swift P, Newbold L, Joint I (2010). The seasonal structure of microbial communities in the Western English Channel. Environ. Microbiol..

[32] Jie GU, Qian CR, Liang HD, Kuang CP, Zuo LM (2017). Seasonal and spatial distribution of chemical oxygen demand(COD) in Caofeidian sea area. Mar. Environ. Sci..

[33] Long AM, Jurgensen SK, Petchel AR, Savoie ER, Brum JR (2021). Microbial ecology of oxygen minimum zones amidst ocean deoxygenation. Front. Microbiol..

[34] Merlo C (2017). Changes in the bacterial community composition of different habitats along a polluted river (Suquía River, Cordoba, Argentina). Ecol. Aust..

[35] Caffrey JM, Bano N, Kalanetra K, Hollibaugh JT (2007). Ammonia oxidation and ammonia-oxidizing bacteria and archaea from estuaries with differing histories of hypoxia. ISME J..

[36] Martiny JB (2006). Microbial biogeography: Putting microorganisms on the map. Nat. Rev. Microbiol..

[37] Wang J (2013). Phylogenetic beta diversity in bacterial assemblages across ecosystems: Deterministic versus stochastic processes. ISME J..

[38] Legendre P, Borcard D, Peres-Neto PR, Legendre P, Borcard D, Peres-Neto PR (2005). Analyzing beta diversity: Partitioning the spatial variation of community composition data. Ecol. Monogr..

[39] Matthew TC, David LK (2003). Part 1 || contribution of major bacterial groups to bacterial biomass production (thymidine and leucine incorporation) in the delaware estuary. Limnol. Oceanogr..

[40] Lee RY, Joye SB (2006). Seasonal patterns of nitrogen fixation and denitrification in oceanic mangrove habitats. Mar. Ecol. Prog. Ser..

[41] Diao M, Huisman J, Muyzer G (2018). Spatio-temporal dynamics of sulfur bacteria during oxic-anoxic regime shifts in a seasonally stratified lake. FEMS Microbiol. Ecol..

[42] Kwon JH, Park HJ, Lee YY (2020). Evaluation of denitrification performance and bacterial community of a sequencing batch reactor under intermittent aeration. J. Environ. Sci. Health Part A Toxic Hazard. Subst. Environ. Eng..

[43] Bolyen ERJ, Dillon MR, Bokulich NA, Abnet CC, Al-Ghalith GA, Alexander H, Alm EJ, Arumugam M, Asnicar F, Bai Y, Bisanz JE, Bittinger K, Brejnrod A, Brislawn CJ, Brown CT, Callahan BJ, Caraballo-Rodríguez AM, Chase J, Cope EK, Da Silva R, Diener C, Dorrestein PC, Douglas GM, Durall DM, Duvallet C, Edwardson CF, Ernst M, Estaki M, Fouquier J, Gauglitz JM, Gibbons SM, Gibson DL, Gonzalez A, Gorlick K, Guo J, Hillmann B, Holmes S, Holste H, Huttenhower C, Huttley GA, Janssen S, Jarmusch AK, Jiang L, Kaehler BD, Kang KB, Keefe CR, Keim P, Kelley ST, Knights D, Koester I, Kosciolek T, Kreps J, Langille MGI, Lee J, Ley R, Liu YX, Loftfield E, Lozupone C, Maher M, Marotz C, Martin BD, McDonald D, McIver LJ, Melnik AV, Metcalf JL, Morgan SC, Morton JT, Naimey AT, Navas-Molina JA, Nothias LF, Orchanian SB, Pearson T, Peoples SL, Petras D, Preuss ML, Pruesse E, Rasmussen LB, Rivers A, Robeson MS, Rosenthal P, Segata N, Shaffer M, Shiffer A, Sinha R, Song SJ, Spear JR, Swafford AD, Thompson LR, Torres PJ, Trinh P, Tripathi A, Turnbaugh PJ, Ul-Hasan S, van der Hooft JJJ, Vargas F, Vázquez-Baeza Y, Vogtmann E, von Hippel M, Walters W, Wan Y, Wang M, Warren J, Weber KC, Williamson CHD, Willis AD, Xu ZZ, Zaneveld JR, Zhang Y, Zhu Q, Knight R, Caporaso JG (2019). Reproducible, interactive, scalable and extensible microbiome data science using QIIME 2. Nat. Biotechnol..

[44] McDonald D (2012). An improved Greengenes taxonomy with explicit ranks for ecological and evolutionary analyses of bacteria and archaea. ISME J..

[45] De Cáceres M, Legendre P, Moretti M (2010). Improving indicator species analysis by combining groups of sites. Oikos.

[46] Robinson MD, McCarthy DJ, Smyth GK (2010). edgeR: A Bioconductor package for differential expression analysis of digital gene expression data. Bioinformatics (Oxf. Engl.).

[47] Clauset A, Newman ME, Moore C (2004). Finding community structure in very large networks. Phys. Rev. E Stat. Nonlinear Soft Matter Phys..

[48] Hartman K (2018). Cropping practices manipulate abundance patterns of root and soil microbiome members paving the way to smart farming. Microbiome.

[49] Douglas GM (2020). PICRUSt2 for prediction of metagenome functions. Nat. Biotechnol..

[50] Segata N (2011). Metagenomic biomarker discovery and explanation. Genome Biol..

[51] Deng WK, Wang YB, Liu ZX, Cheng H, Xue Y (2014). HemI: A toolkit for illustrating heatmaps. PLoS ONE.

[52] Stegen JC (2013). Quantifying community assembly processes and identifying features that impose them. ISME J..

[53] Zhou J, Ning D (2017). Stochastic community assembly: Does it matter in microbial ecology?. Microbiol. Mol. Biol. Rev. MMBR.

[54] Stegen JC, Lin X, Fredrickson JK, Konopka AE (2015). Estimating and mapping ecological processes influencing microbial community assembly. Front. Microbiol..

[55] Chase JM, Kraft NJB, Smith KG, Vellend M, Inouye BD (2011). Using null models to disentangle variation in community dissimilarity from variation in α-diversity. Ecosphere.

