# Supplementary material for: Heterogeneous selection dominated the temporal variation of the planktonic prokaryotic community during different seasons in the coastal waters of Bohai Bay
Source: Sci Rep. 2022 Nov 28;12:20475. doi: 10.1038/s41598-022-24892-1 (PMC9705714; doi:10.1038/s41598-022-24892-1)
Supplement: Supplementary file 2 — Supplementary Information 2. [file 41598_2022_24892_MOESM2_ESM.docx]

Supplementary Material

# Supplementary Figures and Tables


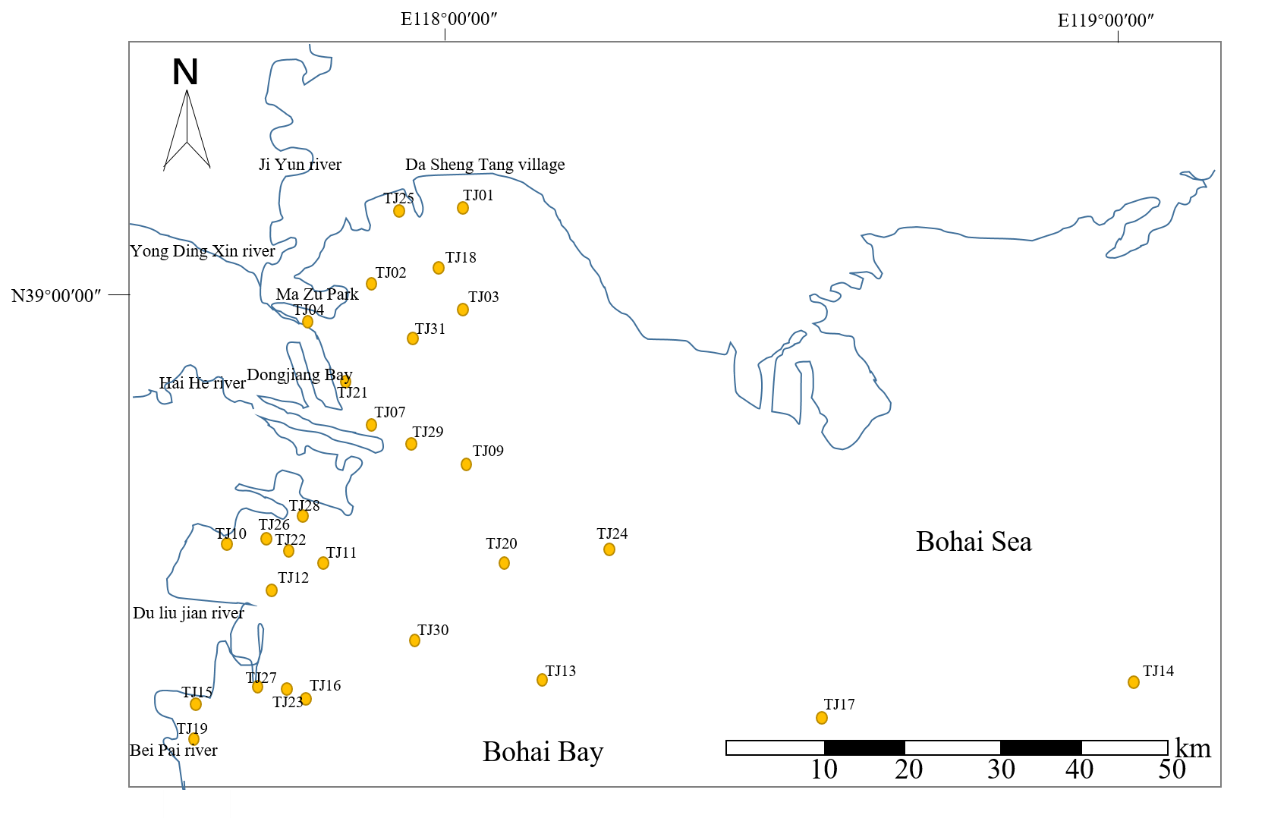


**Figure S1.** Schematic diagram of sampling points in Bohai Bay. In August and Oct, seawater samples from all the 28 stations were sampled and in May seawater samples from 12 stations were samples. Each sample was named after month - site - number of repeats, such as May01_1 means this samples was the first replicate sample from TJ01 in May.


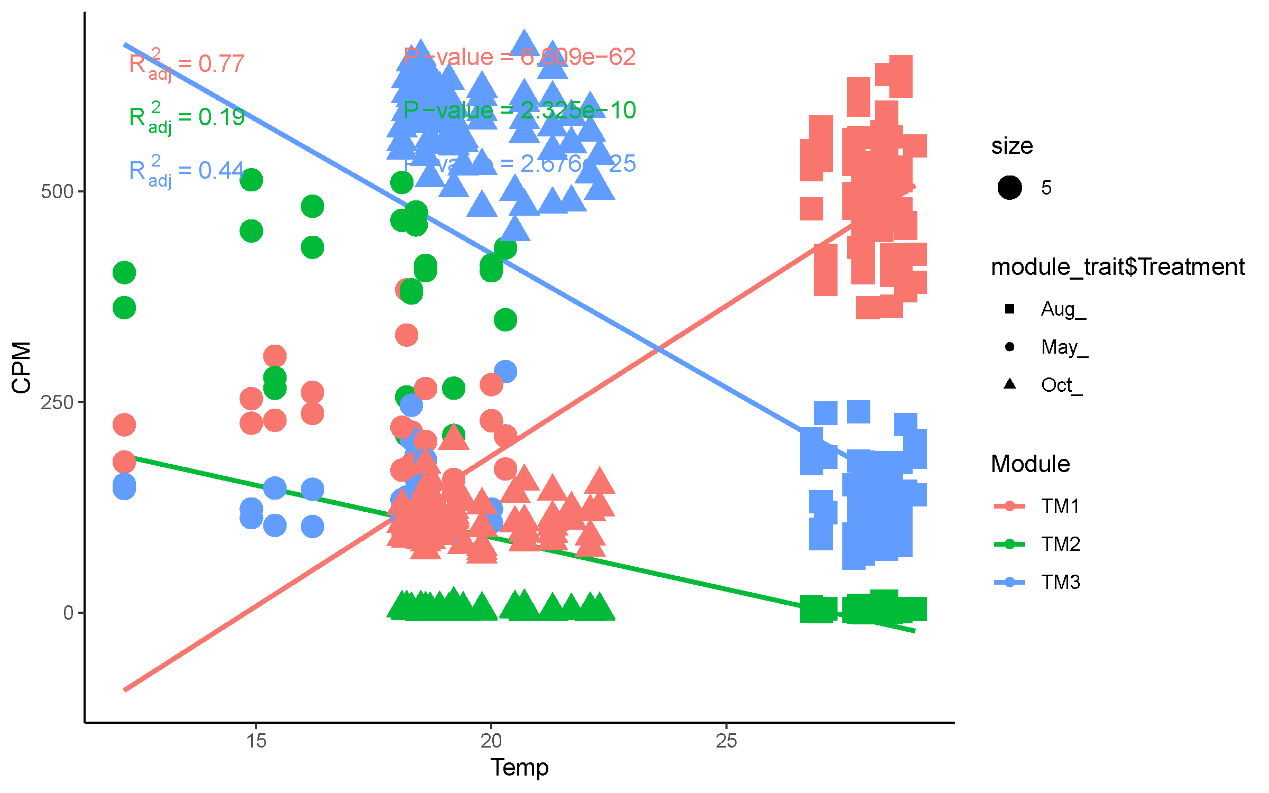


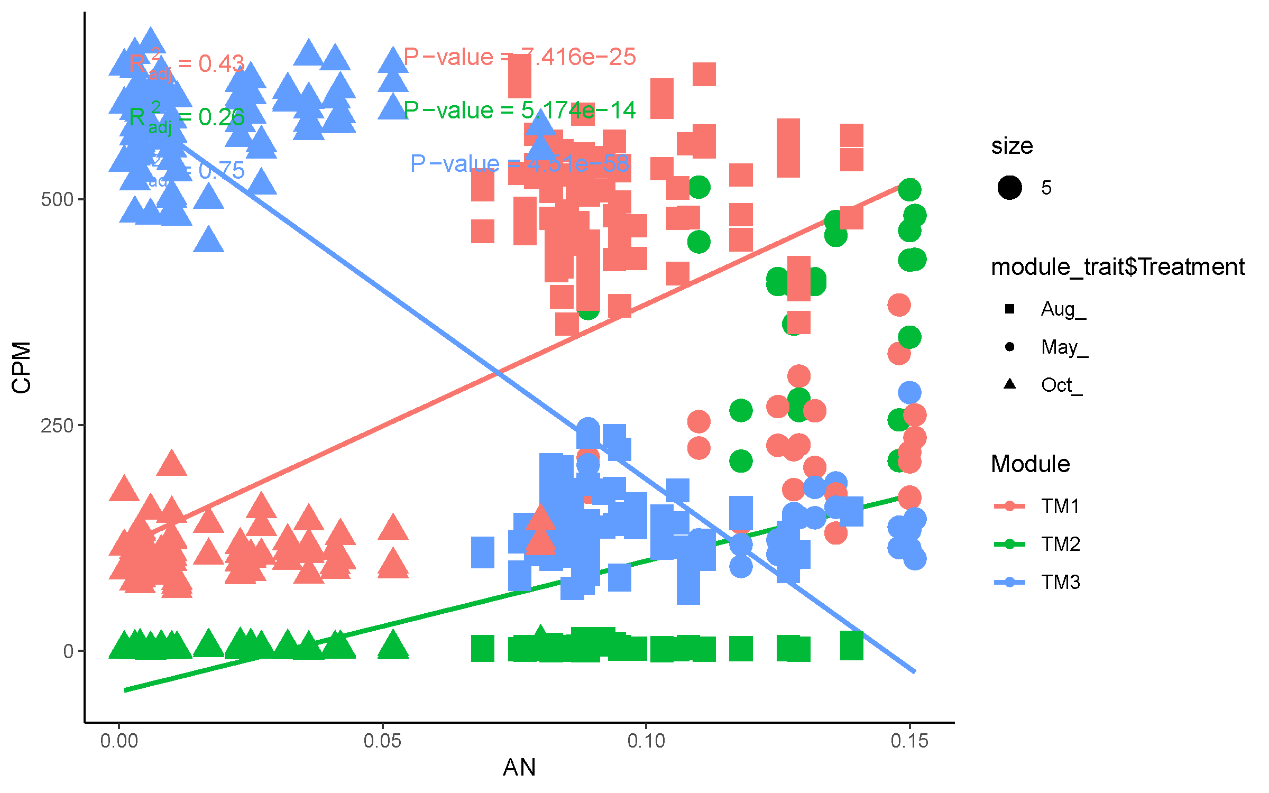


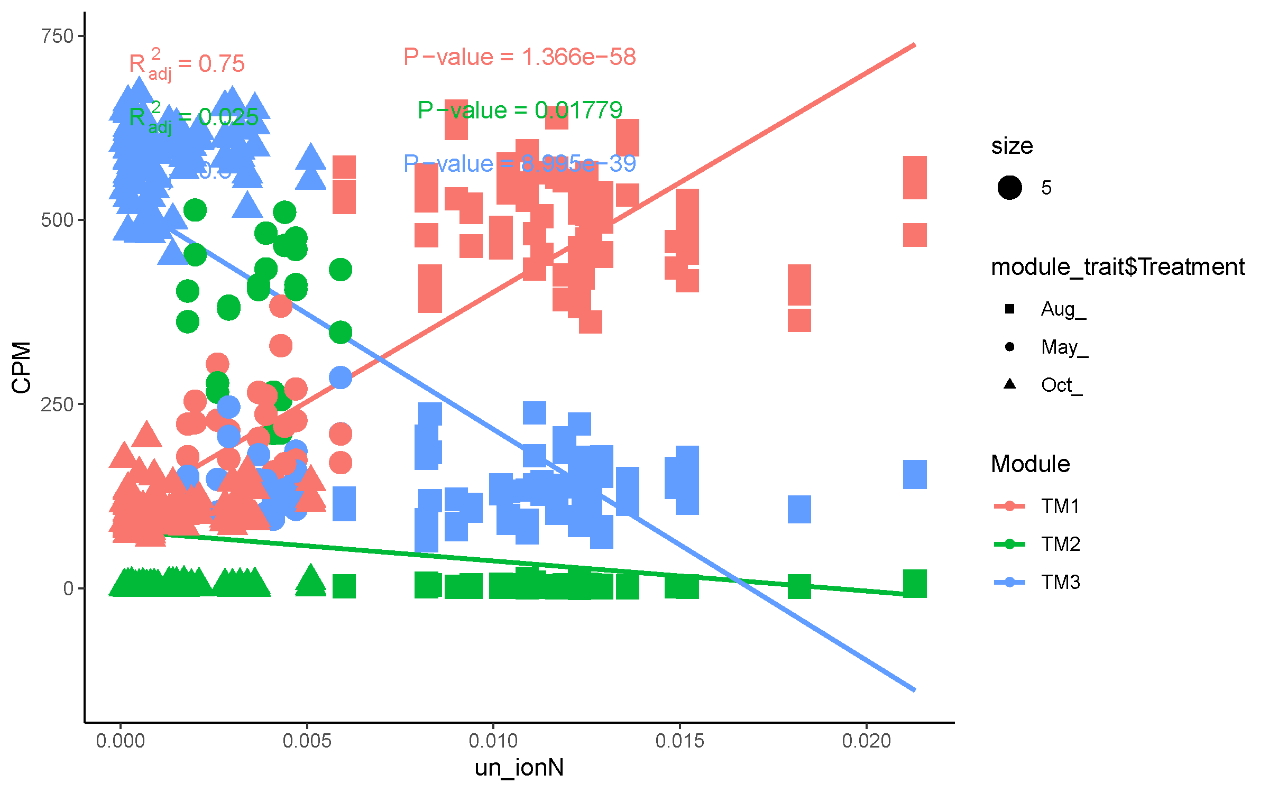


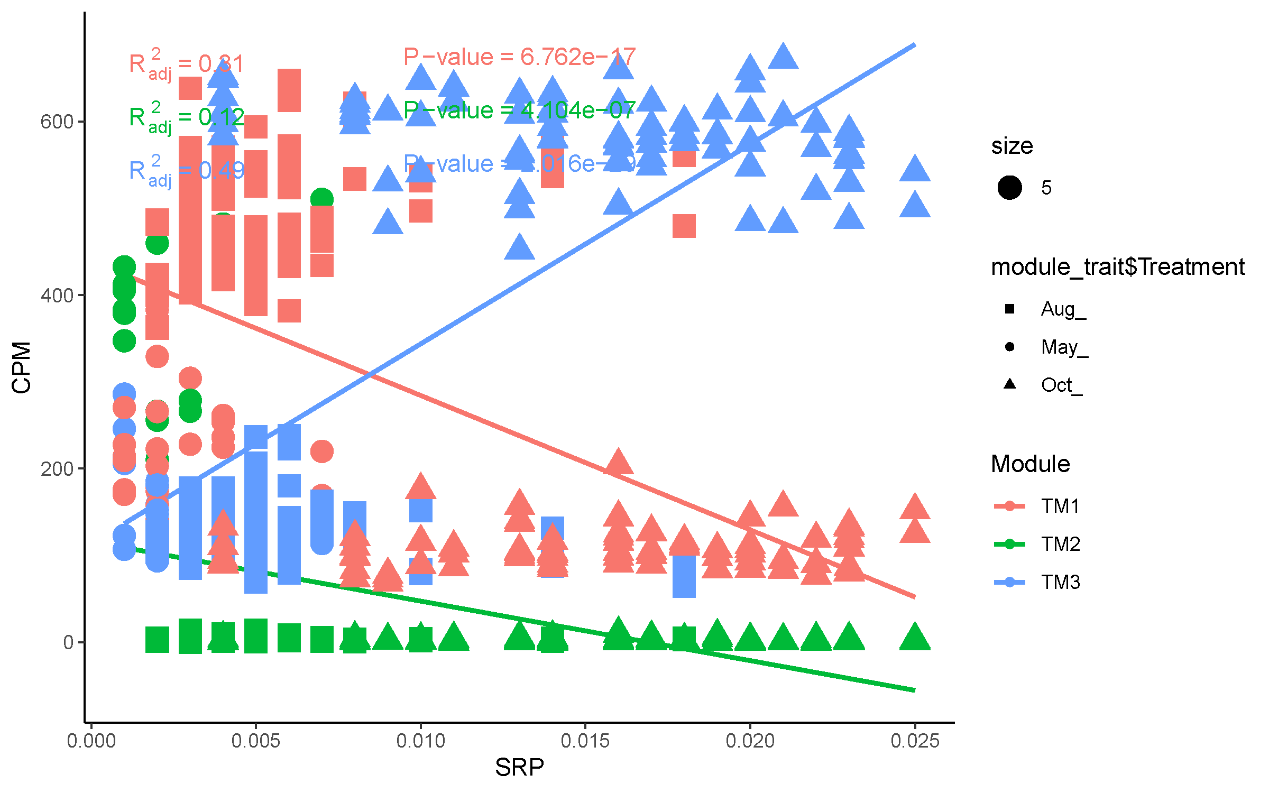


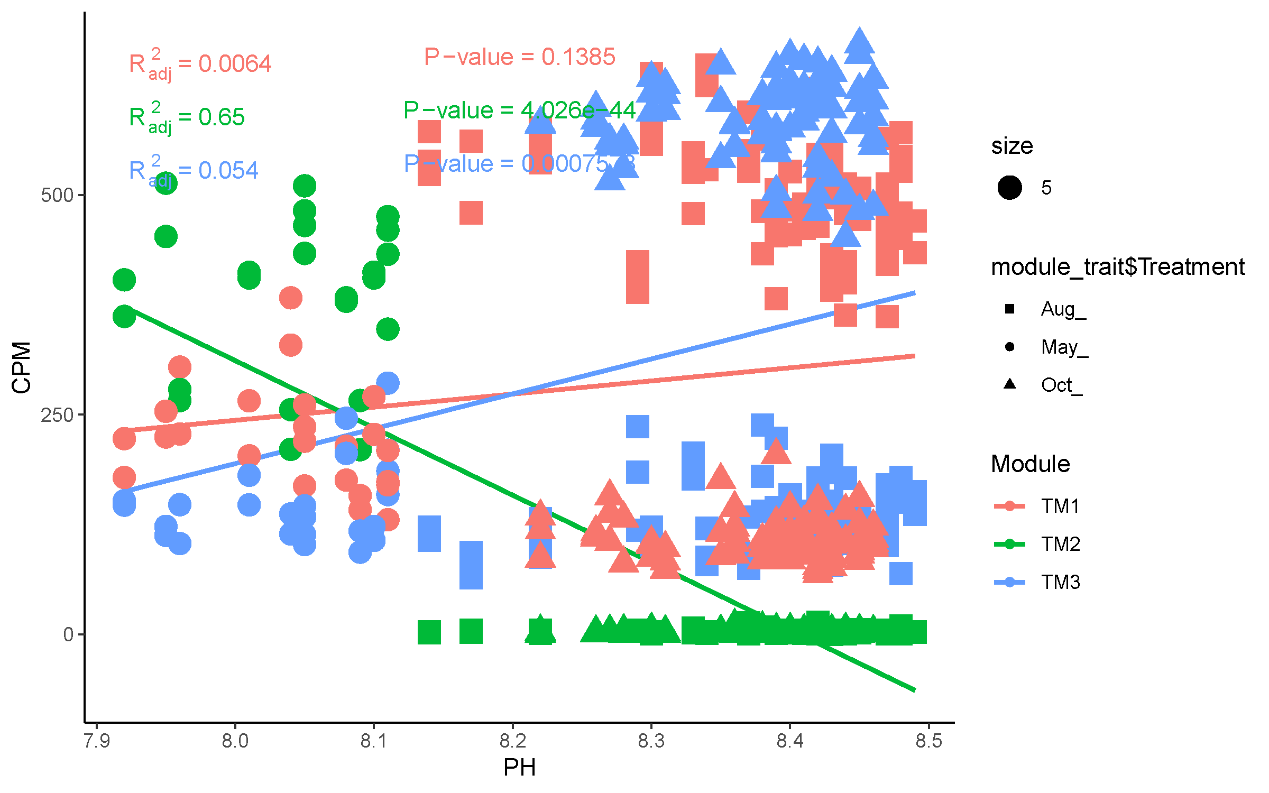


**Figure S2.** Regression between seawater physical and chemical parameters and cumulative relative abundance of network modules.


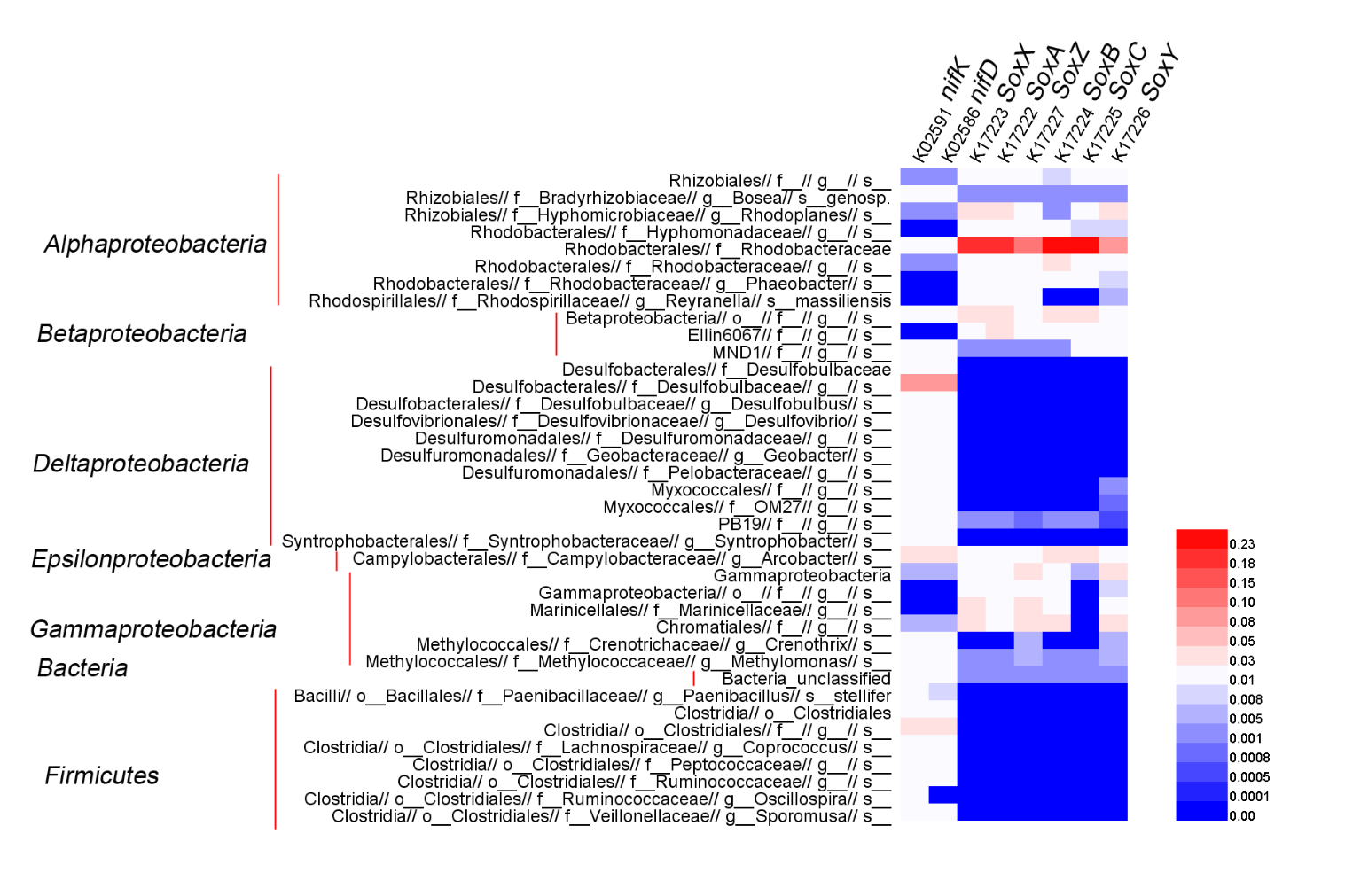
**Figure S3.** The prokaryotic taxa contributed to the significantly varied functional genes related with nitrogen fixation and sulfur oxidation in species level. Prokaryotic gene functions were predicted from 16S rRNA gene-based microbial compositions using the PICRUSt2 algorithm to make inferences from KEGG annotated databases. Value in the heat map represents the contribution percent of the taxa for the functional gene across all samples (phylum *Proteobacteria* is grouped into class level).

**Figure S4.** The prokaryotic taxa contributed to the significantly varied functional genes related with denitrification and sulfate reduction in species level
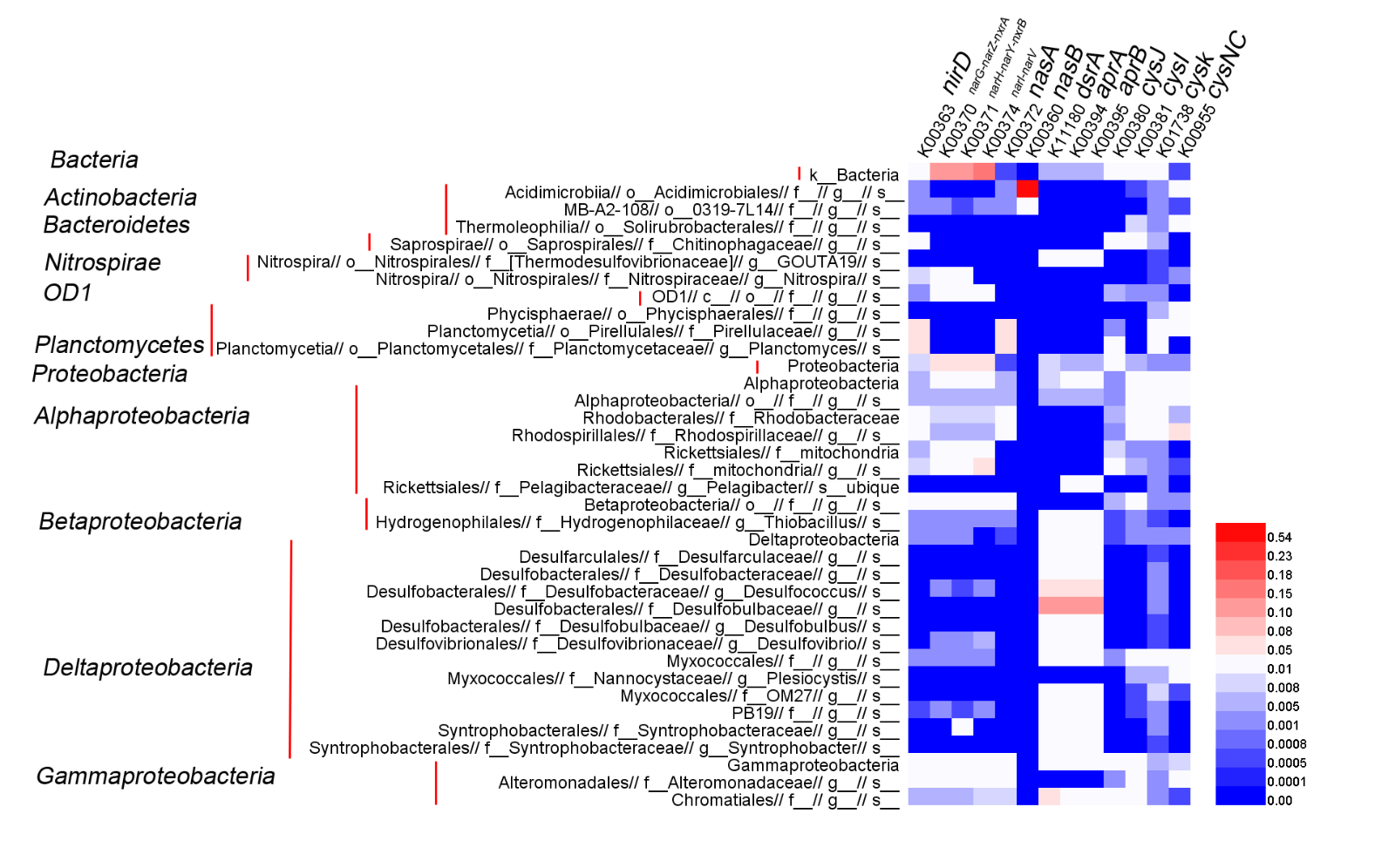
.


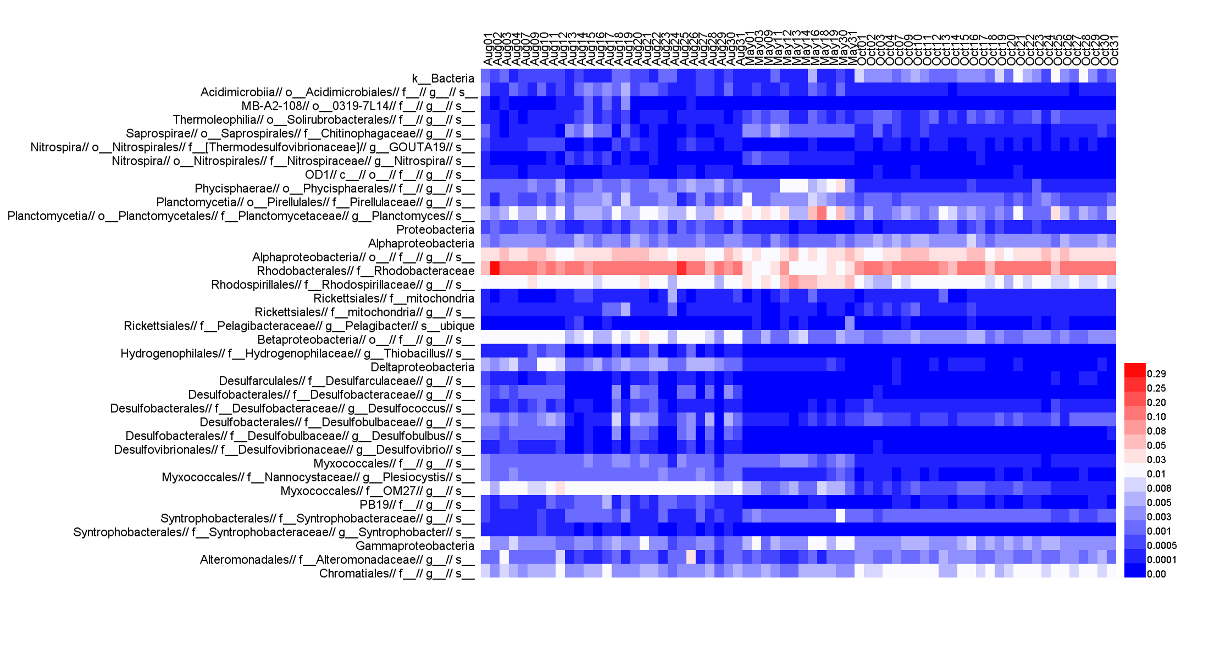


**Figure S5.** The distribution of the taxa which contributed to the functional genes related with denitrification and sulfate reduction. Number in the bar represents the relative abundance of species.

A B

**Figure S6**. Seasonal average rainfall amount over Bohai platform A and Tanggu observe stations. Data is from the national basic meteorological observation station based on the daily precipitation data from 1988 to 2015 of these two observation stations.

**Tables S1** Simple Mantel test demonstrating the correlations of environmental variations (Euclidean distance) or geographic distance

with the variation in PCC (based on weighted/unweighted UniFrac distance, 999 permutations)

| **Variables** | **Weighted_May** | | **Weighted_Aug** | | **Weighted_Oct** | |  | **Unweighted_May** | | | **Unweighted_Aug** | | **Unweighted_Oct** | |
| --- | --- | --- | --- | --- | --- | --- | --- | --- | --- | --- | --- | --- | --- | --- |
|  | **r** | **p** | **r** | **p** | **r** | **p** |  | **r** | | **p** | **r** | **p** | **r** | **p** |
| **pH** | 0.06031 | 0.327 | 0.114 | 0.188 | -0.07572 | 0.73 |  | -0.08193 | 0.681 | | 0.05841 | 0.265 | 0.06793 | 0.272 |
| **AN** | 0.1326 | 0.218 | 0.08015 | 0.257 | -0.08644 | 0.752 |  | 0.03276 | 0.367 | | 0.01379 | 0.393 | -0.05367 | 0.691 |
| **Temp** | 0.1098 | 0.255 | 0.1931 | 0.054 | 0.1342 | 0.111 |  | 0.1696 | 0.182 | | 0.05997 | 0.22 | 0.048 | 0.332 |
| **un_ionN** | 0.1125 | 0.249 | 0.07977 | 0.268 | -0.08461 | 0.745 |  | 0.06507 | 0.332 | | 0.0599 | 0.277 | -0.00081 | 0.509 |
| **COD** | -0.3021 | 0.97 | -0.02606 | 0.562 | -0.03439 | 0.575 |  | -0.05117 | 0.627 | | -0.07661 | 0.768 | 0.04917 | 0.3 |
| **Nitrite_N** | -0.1169 | 0.69 | **0.2821** | 0.007 | **0.2075** | 0.041 |  | 0.03594 | 0.409 | | **0.3174** | 0.002 | 0.04773 | 0.317 |
| **SRP** | **0.5199** | 0.008 | 0.1954 | 0.077 | -0.09405 | 0.831 |  | 0.2676 | 0.098 | | -0.01482 | 0.493 | **0.1427** | 0.048 |
| **Salinity** | 0.1866 | 0.122 | -0.02734 | 0.577 | **0.4582** | 0.001 |  | 0.07835 | 0.297 | | 0.05469 | 0.23 | 0.1275 | 0.089 |
| **DO** | -0.04503 | 0.554 | -0.03103 | 0.57 | -0.2127 | 0.955 |  | 0.174 | 0.187 | | -0.04986 | 0.673 | -0.1676 | 0.921 |
| **DIN** | -0.02373 | 0.531 | 0.05955 | 0.277 | **0.2193** | 0.045 |  | -0.02144 | 0.555 | | -0.03056 | 0.592 | 0.09266 | 0.185 |
| **Chlorophyll_α** | 0.01284 | 0.459 | -0.1031 | 0.788 | **0.2923** | 0.017 |  | 0.1215 | 0.15 | | -0.09625 | 0.856 | 0.1825 | 0.065 |
| **Nitrate_N** | -0.05442 | 0.671 | 0.03779 | 0.35 | **0.2949** | 0.017 |  | 0.08106 | 0.22 | | -0.06295 | 0.741 | 0.1448 | 0.117 |
| **Depth** | 0.04495 | 0.374 | **0.3326** | 0.01 | **0.2581** | 0.029 |  | 0.1617 | 0.18 | | **0.1815** | 0.041 | -0.01758 | 0.553 |
| **Conductivity** | -0.08594 | 0.659 | **0.1893** | 0.04 | -0.01338 | 0.534 |  | -0.01073 | 0.491 | | **0.1853** | 0.032 | 0.1159 | 0.141 |
| **Trans** | 0.2301 | 0.108 | **0.215** | 0.042 | -0.004635 | 0.5 |  | 0.091 | 0.288 | | 0.05884 | 0.245 | 0.03355 | 0.298 |
| **Env** | 0.1142 | 0.28 | 0.2319 | 0.058 | **0.2494** | 0.021 |  | 0.1662 | 0.182 | | 0.1093 | 0.159 | **0.2182** | 0.029 |
| **Geo** | -0.06699 | 0.618 | **0.2487** | 0.034 | **0.3915** | 0.002 |  | -0.1076 | 0.712 | | **0.4597** | 0.001 | 0.07628 | 0.252 |
| **Longitude** | -0.09177 | 0.653 | **0.2378** | 0.038 | **0.2898** | 0.015 |  | -0.06971 | 0.643 | | **0.3637** | 0.002 | -0.03443 | 0.607 |
| **Latitude** | -0.01764 | 0.538 | 0.07272 | 0.199 | **0.2037** | 0.009 |  | -0.05834 | 0.658 | | **0.3455** | 0.001 | **0.169** | 0.02 |

Note: r, correlation coefficients between pairwise distance of each geo-physical-chemical variables and prokaryotic community distance derived from Mantel test with 999 permutations. Data in bold indicate significant correlations (p < .05). Refer to Table 1 and table 2 for variable abbreviations.

**Table S2** Temporal distribution of taxa on phylum level in different sampling month in Bohai Bay.

|  | May | Aug | Oct |
| --- | --- | --- | --- |
| Alphaproteobacteria | 0.4141±0.0907**a** | 0.3417±0.0593**b** | 0.3273±0.0317**b** |
| Gammaproteobacteria | 0.2361±0.0508**b** | 0.2405±0.0593**b** | 0.4423±0.0371**a** |
| Bacteroidetes | 0.1003±0.0342**b** | 0.1398±0.0401**a** | 0.1094±0.0181**b** |
| Planctomycetes | 0.0642±0.0414**a** | 0.0334±0.0179**b** | 0.011±0.0077**c** |
| Verrucomicrobia | 0.0409±0.0516**a** | 0.0508±0.0278**a** | 0.0163±0.0115**b** |
| Betaproteobacteria | 0.0393±0.0218**a** | 0.0342±0.0107**ab** | 0.0301±0.0064**b** |
| Actinobacteria | 0.0386±0.0136**a** | 0.0286±0.0146**b** | 0.0242±0.01**b** |
| Deltaproteobacteria | 0.0192±0.0093**b** | 0.0693±0.0248**a** | 0.0104±0.0056**b** |
| Firmicutes | 0.0148±0.0173**a** | 0.0087±0.0082**b** | 0.0035±0.0045**b** |
| Cyanobacteria | 0.0113±0.0099a | 0.0177±0.0141a | 0.011±0.0061a |
| Acidobacteria | 0.0045±0.0019**a** | 0.003±0.0024**b** | 0.0002±0.0001**c** |
| Gemmatimonadetes | 0.0042±0.0022**a** | 0.0032±0.0058**a** | 0.0004±0.0001**b** |
| Epsilonproteobacteria | 0.0024±0.0045a | 0.0014±0.0022a | 0.0011±0.0018a |
| Chloroflexi | 0.0017±0.0006**b** | 0.0036±0.0023**a** | 0.0001±0.0001**c** |
| TM7 | 0.0016±0.0024**a** | 0.0004±0.0008**b** | 0±0**b** |
| Tenericutes | 0.0016±0.0022**a** | 0.0006±0.0008**b** | 0.0004±0.0002**b** |
| OD1 | 0.0013±0.0041**a** | 0.0002±0.0003**b** | 0±0**b** |
| WPS-2 | 0.0009±0.0018**a** | 0±0**b** | 0±0**b** |
| Bacteria_others | 0.0007±0.0015**b** | 0.0006±0.0004**b** | 0.0044±0.0042**a** |
| PAUC34f | 0.0004±0.0005**a** | 0.0005±0.0004**a** | 0.0001±0.0001**b** |
| Nitrospirae | 0.0004±0.0002**b** | 0.0008±0.0005**a** | 0.0001±0.0001**c** |
| Chlamydiae | 0.0003±0.0005**b** | 0.0007±0.0008**a** | 0.0001±0**b** |
| NKB19 | 0.0003±0.0003**a** | 0.0003±0.0001**a** | 0±0**b** |
| TM6 | 0.0001±0.0003**b** | 0.0025±0.0024**a** | 0.0001±0.0001**b** |
| Chlorobi | 0.0001±0.0002**b** | 0.0007±0.0005**a** | 0.0001±0.0001**b** |
| Lentisphaerae | 0.0001±0.0002**b** | 0.0097±0.0063**a** | 0.002±0.0037**b** |
| Crenarchaeota | 0±0.0001a | 0.0004±0.002a | 0.0034±0.0076a |
| Spirochaetes | 0±0.0001**ab** | 0.0004±0.0008**a** | 0±0**b** |
| Euryarchaeota | 0±0a | 0.0034±0.0087a | 0.0001±0.0003a |
| GN02 | 0±0**b** | 0.0001±0.0001**b** | 0.0008±0.001**a** |
| OP8 | 0±0**b** | 0.0003±0.0003**a** | 0±0**b** |
| Proteobacteria_others | 0±0**c** | 0.0007±0.0005**a** | 0.0003±0.0005**b** |
| SAR406 | 0±0**b** | 0±0.0001**b** | 0.0004±0.0003**a** |

Note: Data in the table are the Means ± SD; The different normal letters in the same row indicate significant difference among sampling month at 0.05 level (n > 3) under Duncan test. Letters in bold means the significant difference of the taxa relative abundance in each sampling month.
